# CT-based radiomics-clinical model for risk assessment of parenteral nutrition-associated hepatic steatosis in chronic intestinal failure and its metabolomic interpretation

**DOI:** 10.3389/fnut.2026.1705520

**Published:** 2026-02-03

**Authors:** Yufei Xia, Ruochen Li, Sirui Liu, Pinwen Zhou, Jiaqi Wang, Xin Qi, Minyi Zhu, Guangming Sun, Xuejin Gao, Li Zhang, Gulisudumu Maitiabula, Xinying Wang

**Affiliations:** 1 Research Institute of General Surgery, Jinling Hospital, Affiliated Hospital of Medical School, Nanjing University, Nanjing, China; 2 Medical School of Southeast University, Nanjing, China; 3 School of Computation, Information and Technology, Technical University of Munich, Munich, Germany

**Keywords:** chronic intestinal failure (CIF), radiomics, multimodal model, metabolomic analysis, steatohepatitis

## Abstract

**Background:**

Patients with chronic intestinal failure (CIF) dependent on parenteral nutrition (PN) are at risk of developing parenteral nutrition-associated hepatic steatosis (PNAHS), a condition that can progress to hepatic fibrosis. Effective methods for predicting PNAHS risk are lacking.

**Methods:**

This retrospective study included 307 CIF patients. Patients were divided into training (*n* = 219) and testing (*n* = 88) sets. Radiomic features (*n* = 1,037 per patient) were extracted from non-contrast abdominal CT scans obtained within 1 week before PN initiation. Clinical characteristics (e.g., demographics, laboratory values) were collected. Multiple predictive models were developed: radiomics, clinical, combined radiomics-clinical, and various deep learning models (e.g., DenseNet121, ResNet18, Unet, etc.). Model performance was evaluated using the area under the receiver operating characteristic curve (AUC), calibration curves, decision curve analysis and Log-rank test. Serum metabolomics analysis was performed to explore biological implications of the model-derived risk scores.

**Results:**

The combined model demonstrated the highest AUC of 0.862 (95% CI: 0.782–0.942) in the testing set, significantly outperforming most other models (*p* < 0.05). Key predictors in the combined model included the radiomics score, total cholesterol level, urea level, PN frequency, and L3-intermuscular fat area. The combined score also demonstrated considerable capability in stratifying patients by duration of PN dependency. The distinct PNAHS risk groups identified by the combined model exhibited significant differences in their metabolic profiles.

**Conclusion:**

We developed a novel predictive model that integrates CT radiomics features with clinical characteristics to effectively predict the risk of PNAHS in patients with CIF and reflects underlying metabolic disturbance.

## 1 Introduction

Intestinal failure (IF) is defined as a state of reduced gut function below the minimum necessary level for the absorption of macronutrients and/or water and electrolytes, requiring intravenous supplementation including parenteral nutrition (PN) to maintain health and/or growth (1). Chronic intestinal failure (CIF) is diagnosed when IF persists for months or years in stable patients, most commonly due to short bowel syndrome (SBS)—a rare disease with an estimated prevalence ranging from 0.4 to 40 per million adults (2, 3).

PN is for a life-sustaining therapy that is critical for maintaining and improving the nutritional status of patients with CIF. However, long-term use of PN is associated with significant complications, among which parenteral nutrition-associated hepatic steatosis (PNAHS) is one of the most concerning (4). PNAHS refers to liver injury related to PN use in the absence of other primary hepatic conditions, such as viral or autoimmune hepatitis, alcohol-related liver damage, or biliary obstruction (5). It may lead to hepatic fibrosis, which is one of the leading causes of mortality in patients with CIF (2).

Notably, not all patients receiving long-term PN treatment develop PNAHS, and the mechanisms underlying this heterogenous responses remain incompletely understood. As many patients with CIF depend on PN in the long term, the accurate prediction of PNAHS risk could significantly improve clinical outcomes by enabling timely interventions and personalized treatment (6). However, predicting PNAHS at an early stage, before overt abnormalities manifest in serum biomarkers or medical imaging, remains a substantial clinical challenge.

Radiomics, a technique for extracting high-dimensional quantitative features from medical images, has shown promise for predicting various liver diseases, including non-alcoholic fatty liver disease (NAFLD) (7–10). In recent years, deep learning has emerged as a powerful tool for medical image analysis, capable of enhancing predictive accuracy through automated feature extraction and pattern recognition. Nevertheless, challenges such as poor interpretability and a propensity for overfitting, particularly with small sample sizes, persist (11, 12). Comparative studies of deep learning and traditional radiomics models have reported inconsistent results, underscoring the need for further investigation in specific clinical contexts (13–15). To date, no studies have applied radiomics or deep learning techniques to predict the risk of PNAHS, a rare disease that shares some pathological features with NAFLD. Thus, the development of predictive models for PNAHS risk utilizing these advanced technologies is both warranted and clinically significant.

This study aimed to develop the first predictive model for PNAHS risk assessment by integrating radiomics features from pre-PN non-contrast CT scans with clinical characteristics in patients with CIF. We also compared the performance of this model against various deep learning architectures. Furthermore, we aimed to provide a metabolic interpretation for the combined predictive model through serum metabolomics profiling.

## 2 Materials and methods

### 2.1 Study sample

This retrospective study initially screened 994 adult patients with CIF from the General Surgery Department of Jinling Hospital between January 2010 and August 2023. The inclusion criteria were: (1) age 18–80 years; (2) diagnosis of intestinal failure with an inability to maintain weight via enteral nutrition alone, necessitating PN at least weekly for 3 months; (3) availability of long-term clinical data, including hematological tests and nutritional support records; and (4) availability of an abdominal non-contrast CT scan performed within 1 week prior to PN initiation. Exclusion criteria were: (1) did not receive long-term PN; (2) had pre-existing liver abnormalities on imaging or persistent, unexplained elevations of liver enzymes and bilirubin prior to PN initiation; (3) had poor-quality CT images or insufficient clinical data; (4) underwent major abdominal surgery during PN treatment; (5) had a diagnosis of active malignancy; and (6) had conditional known to significantly affect liver function, including sepsis, chronic glucocorticoid use, hepatitis B surface antigen (HBsAg) positivity, a history of heavy alcohol consumption, or chronic kidney disease.

Since liver biopsies are seldom performed in patients with CIF in clinical practice and studies, the diagnosis of PNAHS was based on biochemical and imaging criteria rather than histology (16–19). Therefore, with reference to the diagnostic criteria for PNAHS from the European Society for Clinical Nutrition and Metabolism (ESPEN) position paper, we defined PNAHS as the presence of at least one of the two core criterion as follows for ≥2 consecutive weeks in patients receiving PN for >3 months:

1. AST/ALT ratio index (AARI): AST/ALT <1 when both AST and ALT levels exceed the upper limit of normal (ULN);
2. Ultrasound criterion: hepatic steatosis diagnosed by ultrasound according to the European Association for the Study of the Liver (EASL) guidelines (5).

According to the ESPEN criteria, the following supporting features—suggestive of other types of PN-associated liver diseases—may coexist but are not required for the PNAHS diagnosis:

1. Total bilirubin >1 mg/dL (>17.1 μmol/L) and direct bilirubin >0.3 mg/dL;
2. Total bilirubin >3 mg/dL plus elevated direct bilirubin, thrombocytopenia, and splenomegaly;
3. Imaging/pathology-confirmed portal hypertension, hepatic fibrosis, or cirrhosis;
4. ALP, GGT, and direct bilirubin >1.5 × ULN;
5. APRI >0.88 or Fibrosis 4 Score (FIB-4) ≥ 2.67 (5).

**Figure 1:**
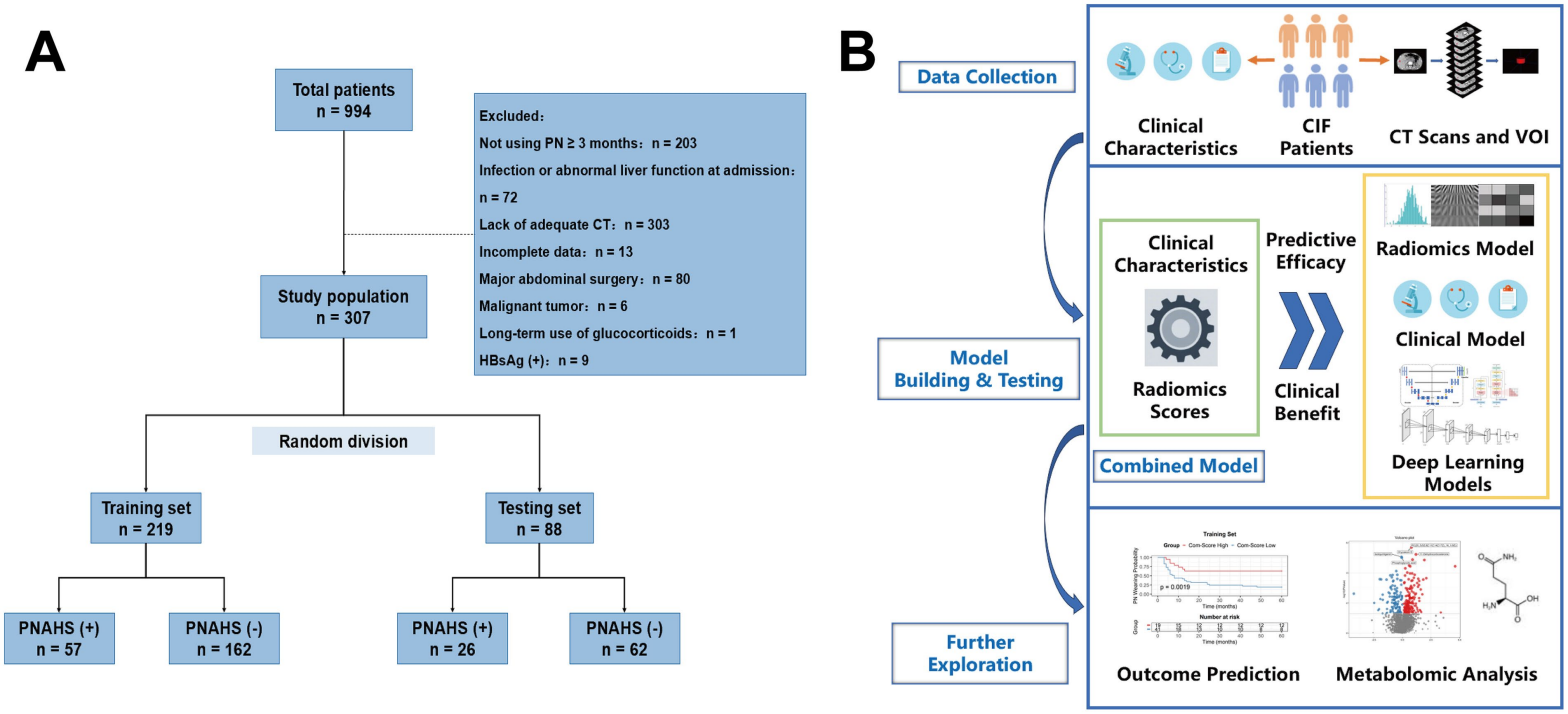
**(A)** Flow diagram of the patient selection process. **(B)** Workflow of the study. PHAHS, parenteral nutrition-associated hepatic steatosis; CIF, chronic intestinal failure; VOI, volumes of interest.

All patients included in the study were randomly allocated to a training set and an independent testing set in a 7:3 ratio (Figure 1A).

### 2.2 Clinical characteristics, radiologic assessment and metabolomic data

All the data was retrospectively collected or derived from electronic medical records and follow-up archives. Specifically, the following variables were collected:

1. Demographics at PN initiation: Age, sex and body mass index (BMI).
2. Medical history: Type of SBS, hypertension, diabetes and thrombosis.
3. Nutritional status at PN initiation: Resting energy expenditure (REE) measured by indirect calorimetry, Nutrition Risk Screening 2002 (NRS2002) score (20), and diagnosis OF severe malnutrition according to Global Leadership Initiative on Malnutrition (GLIM) criteria (21).
4. Laboratory parameters at PN initiation: Alanine aminotransferase (ALT), aspartate aminotransferase (AST), gamma-glutamyl transferase (GGT), alkaline phosphatase (ALP), total bilirubin (TBIL), direct bilirubin (DBIL), indirect bilirubin (IBIL), creatinine, urea, triglycerides (TG), and total cholesterol (TC).
5. PN-related characteristics: Glucose-to-lipid ratio and the proportion of fish oil in the lipid emulsion in the PN formula, and frequency of PN use. These characteristics reflect the stable PN regimen designed according to the ESPEN guidelines after initial adjustments.
6. Radiological features from CT: Intramuscular fat area and skeletal muscle area at the third lumbar vertebra (L3) cross-section. The L3 skeletal muscle index (SMI, cm^2^/m^2^) was calculated by normalizing the skeletal muscle tissue area to height squared. According to Japan Society of Hepatology guidelines, sarcopenia was defined as an L3 SMI of <42 cm^2^/m^2^ for men and <38 cm^2^/m^2^ for women (22);
7. Follow-up records: The weaning status from PN and time to PN independence, recorded for up to 5 years after discharge.
8. Metabolomic data: Serum samples for metabolomic analysis were collected during routine blood tests performed within 1 week before PN initiation.

### 2.3 Radiomics feature extraction

The CT Study Protocols are presented in Appendix S1.1. After reviewing the literature, the volumes of interest (VOIs) selected in this study was the right anterior lobe of the liver (right branch of the portal vein) centered at the umbilical portion of the portal vein, encompassing a total of 10 slices with a diameter of 2 cm, avoiding visible vessels, liver fissures, and cysts (23). In total, 1,037 radiomics features were extracted for each case and 792 features with an intraclass correlation coefficient (ICC) above 0.75 were included in the radiomics model developing. Feature types, extracted features and the ICC of each feature are presented in Supplementary Table S1.

### 2.4 Model building and testing

The detailed process of radiomics model building can be found in the Appendix S1.2, Supplementary Table S2. All processes were implemented using FeAture Explorer Pro (FAE, V 0.5.12) on Python (3.7.6). In the training set, univariate and multivariate logistic regression were employed to identify independent risk factors for PNAHS, thereby constructing a clinical predictive model and a combined (radiomics-clinical) model. To comprehensively evaluate the performance of the radiomics and deep learning models in medical imaging analysis, we developed nine deep learning models using Medical Open Network for AI (MONAI, V 1.6.dev2548) on Google Colaboratory,^1^ including classical convolutional neural networks (DenseNet121 and ResNet18), models tailored for image segmentation (Unet and SwinUNETR), emerging transformer-based architectures (Vision Transformer, VIT), and a hybrid model integrating deep learning features and machine learning (ResNet features with XGBoost/FC algorithm). Additionally, a multilayer perception (MLP) and basic convolutional neural network (CNN) were included for comparison. The code for model building can be found in GitHub.^2^ Each model was integrated with the same clinical variables included in the final radiomics-clinical model. All models were validated in a 5-fold cross validation and tested in the same testing set.

### 2.5 Outcome prediction and biologic functions exploration

The radiomics score and the combined score of patients were calculated. The formulas can be seen in Appendix S2. A total of 122 patients were followed up until they weaned off PN or 5 years after the initiation of PN treatment; however, 44 patients with type I SBS were excluded before the clinical outcome prediction, because most of them had undergone ostomy reversal surgery 6 months after the initial surgery, which could potentially affect their clinical outcomes. Patients were divided into high-risk and low-risk groups based on the median value of the radiomics score (training set: −0.208, testing set: −0.086, *p* > 0.05) and the combined score (training set: −2.006, testing set: −1.932, *p* > 0.05) in the training and testing sets. The log-rank test was used to compare the PN dependency between the low- and high-score groups. We also compared the differences in metabolites and metabolic pathways between correctly predicted patients in different risk groups categorized by the combined score. The details of the metabolomic analysis can be found in Appendix S3.

### 2.6 Statistical analysis

According to previous reports, the incidence of PNAHS varies between 0 and 50% in different reports (24). Assuming a 25% incidence rate of PNAHS in our center’s patients with CIF, a sample size of at least 288 patients was needed considering a 5% two-tailed Type I error rate and 80% power of the test (calculated by PASS version 15.0.5, NCSS, USA). Continuous variables are reported as medians and IQRs, while categorical variables are reported as numbers and percentages. The Mann–Whitney *U* test was used to analyze continuous data and the chi-square test was used for the analysis of categorical variables. Univariate and multivariate logistic regression analyses were used to determine the odds ratios (ORs) and 95% confidence intervals for risk factors. The discriminative performance of the models predicting PNAHS risk was studied using AUC and compared using the Delong test, calibration curves, and decision curve analysis. Accuracy, sensitivity and specificity were also calculated at a cutoff value that maximized the value of the Yorden index. Correlation analysis was performed using multiple linear regression. Regression coefficients (Beta) were adjusted for sex, age, BMI, medical history (hypertension, diabetes, and thrombosis), and GLIM nutritional status. A Beta value was considered statistically significant if the corresponding *p*-value was < 0.05. Statistical analysis was conducted using IBM SPSS Statistics (version 29.0.1.0) and R software (version 4.2.2). Differences were considered statistically significant at *p* < 0.05.

## 3 Results

### 3.1 Characteristics of the study sample

Among the 994 patients with intestinal failure, 307 were included in this study after exclusion (Figure 1A). The average age of the included patients was 52.0 (43.0–60.0) years old, with a predominance of males (*n* = 212, 69.0%). SBS was the most common cause of intestinal failure (*n* = 289, 94.1%), with type I in 84 cases (29.1%), type II in 106 cases (36.7%), and type III in 99 cases (34.2%). Among all patients, 83 (27.0%) were diagnosed with PNAHS during PN treatment. All patients were randomly divided into training and testing sets in a 7:3 ratio, with no significant differences in baseline characteristics between the two sets (Supplementary Table S3). In both sets, differences were observed in TC level, creatine level, urea level, PN frequency and L3-intramuscular fat area between patients who developed PNAHS during PN treatment and those who did not, and age was also significantly different between them in the testing set (Supplementary Table S4).

### 3.2 Building and performance testing of radiomics model, clinical model, deep learning models and combined model for PNAHS prediction

The study workflow is illustrated in Figure 1B. The radiomics model developed from 4 features achieved the highest Yorden index (0.600) in 5-fold cross-validation. The coefficients of these features are detailed in Supplementary Table S2, and their distributions across different sets and outcome groups are demonstrated in Supplementary Figure S1.

**Figure 2:**
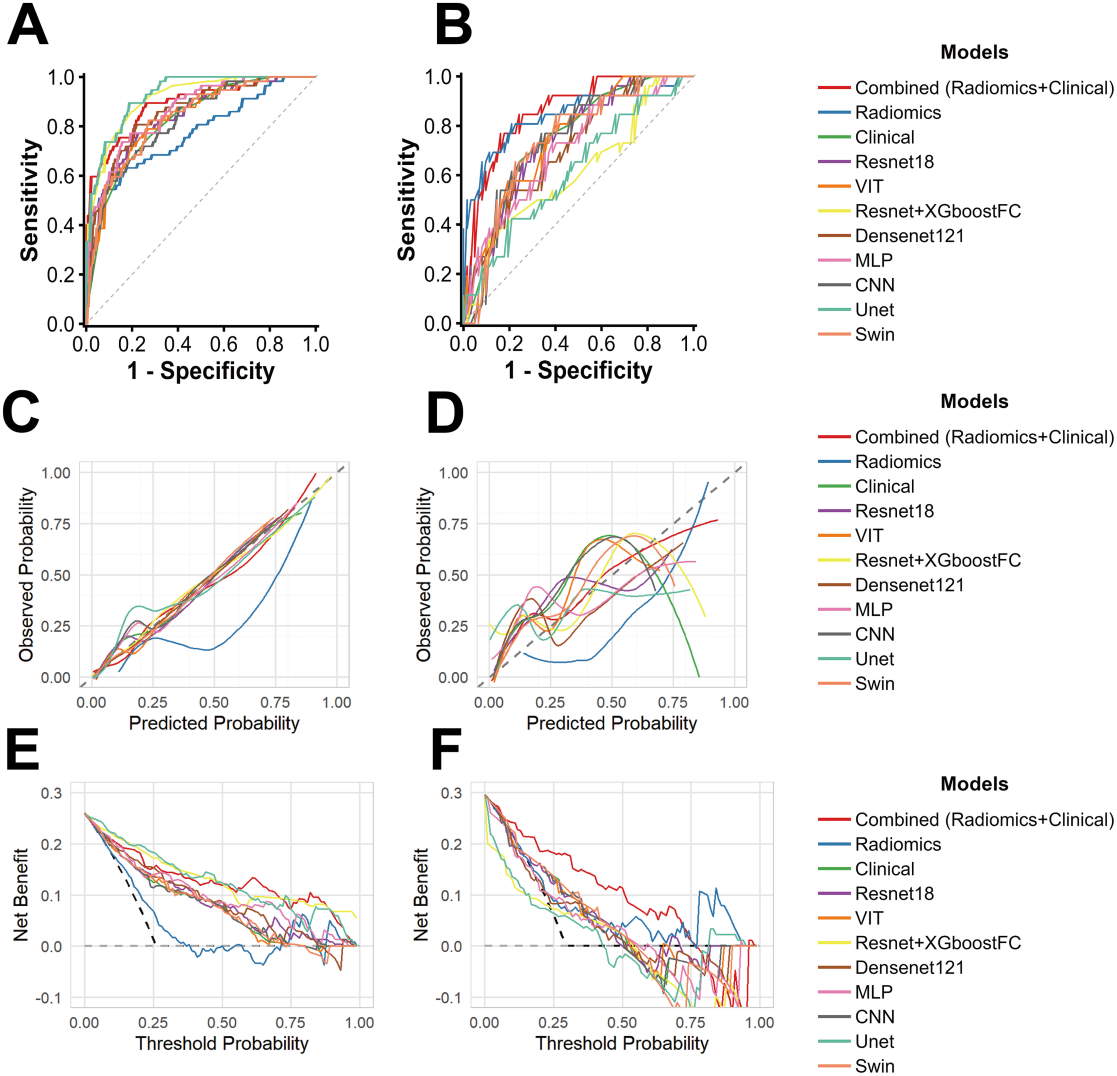
Performance of predictive models. **(A)** Receiver operating characteristic (ROC) curves of various models in the training set (*n* = 219). **(B)** ROC curves of various models in the testing set (*n* = 88). **(C,D)** Calibration curves for the predictive models in the training set and testing set, respectively. The *x*-axis represents the predicted probability of high PNAHS risk and the *y*-axis represents the observed probability. The dotted line shows the ideal calibration (prediction = observation) and solid lines indicate model performance. **(E,F)** Decision curve analysis for the predictive models in the training set and testing set, respectively. The *x*-axis represents the threshold probability and the *y*-axis represents the net benefit. Lines represent net benefit of models at varying thresholds, compared to all-patient treatment or no treatment.

Among all the collected clinical characteristics and the radiomics score, univariable analysis of the training set identified sex, TC level, creatine level, urea level, frequency of PN, L3-intermuscular fat area and radiomics score as risk factors for PNAHS (*p* < 0.05). Subsequent multivariable analysis confirmed TC level, urea level, PN frequency, L3-intermuscular fat area and the radiomics score as independent predictors for PNAHS (*p* < 0.05) (Table 1). These variables were used to build the clinical and combined model. Sex and serum creatine level were excluded due to the insignificant impact in the forward selection. The performance metrics of all models are summarized in Tables 2, 3, with corresponding ROC curves presented in Figures 2A,B. In the training set, the combined model demonstrated a significantly higher AUC than most other models, including the radiomics model, clinical model, and various deep learning models such as VIT and SwinUNETR. Notably, no deep learning model significantly outperformed the combined model in terms of AUC. While performance differences between models appeared less pronounced during 5-fold cross-validation (Supplementary Table S5), the combined model’s superiority was evident in the independent testing set, where its AUC [0.862 (95% CI: 0.782–0.942)] was significantly higher than all other models except the radiomics model (*p* < 0.05).

**Table 1:** Logistics regression analysis of variables for their association with PNALD in patients in the training set.

Variables	Univariable analysis		Multivariable analysis	
	OR	*p*-value	OR	*p*-value
Sex (male vs. female)	2.90 (1.38, 6.69)	0.008	NA	NA
Age (≤50 vs. > 50 years)	1.67 (0.91, 3.13)	0.102	/	/
BMI (18.5–23.9 vs. < 18.5 & > 23.9 kg/m^2^)	1.12 (0.61, 2.09)	0.707	/	/
REE (≤1,300 vs. > 1,300 kcal/d)^a^	1.04 (0.57,1.90)	0.899	/	/
NRS-2002 (< 5 vs. ≥ 5)	0.84 (0.45, 1.55)	0.592	/	/
GLIM-severe malnutrition (non- or mild vs. severe)	1.31 (0.71 2.43)	0.392	/	/
SBS types (I vs. II and III)*	1.04 (0.71, 1.53)	0.834	/	/
Underlying diseases				
Hypertension (absent vs. present)	1.69 (0.86, 3.23)	0.120	/	/
Diabetes (absent vs. present)	1.10 (0.43, 2.55)	0.837	/	/
Thrombosis (absent vs. present)	1.10 (0.58, 2.03)	0.770	/	/
ALT (≤40 vs. >40 U/L)^b^	1.16 (0.57, 2.29)	0.665	/	/
AST (≤35 vs. >35 U/L)^b^	1.05 (0.47, 2.22)	0.897	/	/
ALP (≤135 vs. >135 U/L)^b^	1.70 (0.78, 3.58)	0.169	/	/
GGT (≤45 vs. >45 U/L)^b^	1.27 (0.61, 2.56)	0.508	/	/
TBIL (≤23 vs. >23 μmol/L)^b^	1.39 (0.54, 3.34)	0.471	/	/
DBIL (≤6.8 vs. >6.8 μmol/L)^b^	0.72 (0.35, 1.43)	0.367	/	/
IBIL (≤16.2 vs. >16.2 μmol/L)^b^	1.45 (0.37, 4.81)	0.555	/	/
Creatinine (≤73 vs. >73 μmol/L)^b^	3.36 (1.81, 6.37)	<0.001	NA	NA
Urea (≤7.5 vs. >7.5 mmol/L)^b^	5.66 (2.92, 11.54)	<0.001	4.80 (2.11, 11.599)	<0.001
TG (≤1.7 vs. >1.7 mmol/L)^b^	1.52 (0.82, 2.81)	0.183	NA	NA
TC (≤5.18 vs. >5.18 mmol/L)^b^	4.24 (1.41, 13.46)	0.010	4.50 (1.09, 19.21)	0.038
Ratio of glucose to lipid of PN (≥1.5 vs. <1.5)^c^	0.55 (0.28, 1.06)	0.072	NA	NA
Ratio of fish oil to lipid of PN (≥0.1 vs. <0.1)^c^	1.73 (0.94, 3.20)	0.078	NA	NA
Frequency of PN (≤3 vs. >3 d/w)	3.18 (1.58, 6.87)	0.002	4.76 (1.90, 13.23)	0.001
L3-muscle area (≤92 vs. > 92 mm^2^)^a^	0.74 (0.40, 1.36)	0.339	NA	NA
L3-intermuscular fat area (≤648 vs. > 648 mm^2^)^a^	5.46 (2.79, 11.30)	<0.001	4.38 (2.03, 10.01)	0.002
Sarcopenia (absent vs. present)	0.87 (0.41, 1.96)	0.732	NA	NA
Radiomics score	10.07 (5.06, 21.80)	<0.001	1.87 (1.41, 12.55)	<0.001

^a^The classification threshold is the median of this indicator among all the patients. ^b^The classification threshold depends on the normal values of various indicators provided by the testing department of this center. ^c^The classification threshold was determined with reference to the ESPEN guidelines. “NA” was filled in the table when a variable showed a *p* value > 0.05 in the univariable analysis but not in the multivariable analysis. *18 cases have missing data.

**Table 2:** Performance of prediction models for predicting the PNALD in patients with CIF in the training set.

Model	Sensitivity	Specificity	Accuracy	Youden index	AUC (95% CI)	*p*-value^a^
Combined model	0.895	0.740	0.781	0.635	0.889 (0.836, 0.942)	NA
Radiomics model	0.632	0.827	0.776	0.459	0.776 (0.699, 0.852)	<0.001
Clinical model	0.754	0.728	0.735	0.483	0.826 (0.767, 0.885)	0.010
Unet	0.894	0.815	0.836	0.710	0.927 (0.894, 0.960)	0.102
ResNet+XGboost/FC	0.859	0.802	0.817	0.662	0.874 (0.875, 0.954)	0.327
VIT	0.789	0.741	0.753	0.530	0.834 (0.775, 0.892)	<0.001
SwinUNETR	0.824	0.728	0.753	0.553	0.832 (0.773, 0.892)	<0.001
DenseNet121	0.807	0.784	0.790	0.591	0.856 (0.801, 0.912)	0.151
ResNet18	0.772	0.778	0.776	0.550	0.840 (0.780, 0.899)	0.036
CNN	0.702	0.784	0.762	0.486	0.824 (0.764, 0.884)	0.008
MLP	0.737	0.846	0.817	0.582	0.864 (0.811, 0.916)	0.328

^a^The AUC of the combined model was compared with that of the other models using Delong test. Differences were considered statistically significant at *p* < 0.05.

**Table 3:** Performance of prediction models for predicting the PNALD in patients with CIF in the testing set.

Model	Sensitivity	Specificity	Accuracy	Youden index	AUC (95% CI)	*p*-value^a^
Combined model	0.769	0.839	0.818	0.608	0.862 (0.782, 0.942)	NA
Radiomics model	0.808	0.790	0.795	0.598	0.842 (0.738, 0.947)	0.657
Clinical model	0.654	0.758	0.727	0.412	0.750 (0.646, 0.855)	0.003
Unet	0.423	0.806	0.693	0.230	0.620 (0.490, 0.750)	<0.001
ResNet+XGboost/FC	0.346	0.887	0.727	0.233	0.610 (0.478, 0.742)	<0.001
VIT	0.769	0.645	0.681	0.414	0.750 (0.644, 0.856)	0.003
SwinUNETR	0.846	0.597	0.670	0.443	0.741 (0.632, 0.850)	0.001
DenseNet121	0.538	0.806	0.727	0.345	0.713 (0.600, 0.826)	0.001
ResNet18	0.731	0.693	0.704	0.424	0.748 (0.641, 0.854)	0.001
CNN	0.769	0.661	0.693	0.430	0.743 (0.635, 0.851)	0.002
MLP	0.731	0.613	0.648	0.344	0.710 (0.592, 0.827)	0.001

^a^The AUC of the combined model was compared with that of the other models using Delong test. Differences were considered statistically significant at *p* < 0.05.

Assessment of model calibration revealed that that although most models showed good calibration with the observed probabilities in the training set, with deep learning models calibrating better than the radiomics model. However, in the testing set, only the combined model maintained close calibration across the probability spectrum (Figures 2C,D). Its Brier score was also the lowest (0.139; Supplementary Tables S6, S7). In contrast, most deep learning models systematically overestimated the risk at higher prediction scores in the testing set, while the radiomics model exhibited a tendency to underestimate the risk at lower scores in both sets (Figures 2C,D). This trend was corroborated by decision curve analysis, where the combined model provided the highest clinical benefit (Figures 2E,F). The predictors of the combined model were presented as a nomogram and a forest plot to facilitate clinical interpretation and individualized and illustrate the contribution of each variable (Supplementary Figure S2).

### 3.3 Outcome prediction and biologic functions exploration

**Figure 3:**
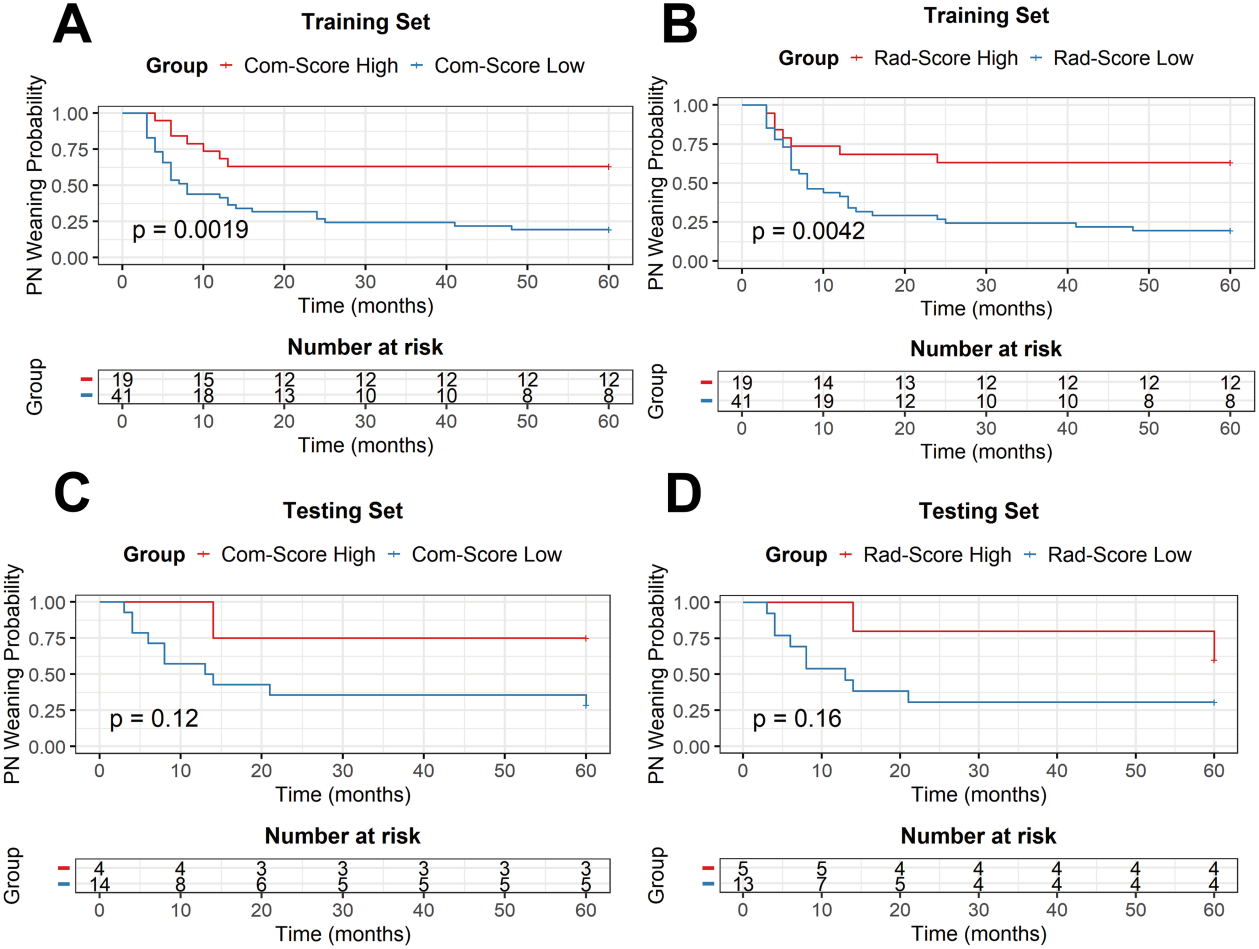
Kaplan–Meier curves of PN dependency of patients with type II and type III SBS in the training and testing sets. **(A,B)** Represent the training set where the median radiomics scores and combined scores were used, respectively, to divide the patients into high- and low-risk groups, and **(C,D)** Represent the testing set. The x-axis represents time (in months) from diagnosis to PN independence or censoring, and the y-axis represents the number of patients dependent on PN.

Followed up data were available for 60 patients in the training set and 18 in the testing set were followed up, with observation continuing until either PN discontinuation or for up to 5 years after PN initiation. Overall, 51 patients achieved PN independence, with a median PN dependency duration of 6 (4–13) months. The 1-, 2- and 5-year PN wean-off rate were 46.1, 60.2 and 65.4%, respectively. Both the combined score and the radiomics score were significantly associated with PN dependency in the training set (*p* < 0.01) (Figures 3A,B). A similar trend was observed in the testing set, where the models showed differential predictive ability, particularly in the early phase of follow-up, although this difference did not reach statistical significance over the entire 5-year period (Figures 3C,D). Among the 18 patients followed in the testing set, nearly all who remained PN-dependent during the first 2 years failed to achieve independence thereafter.

**Figure 4:**
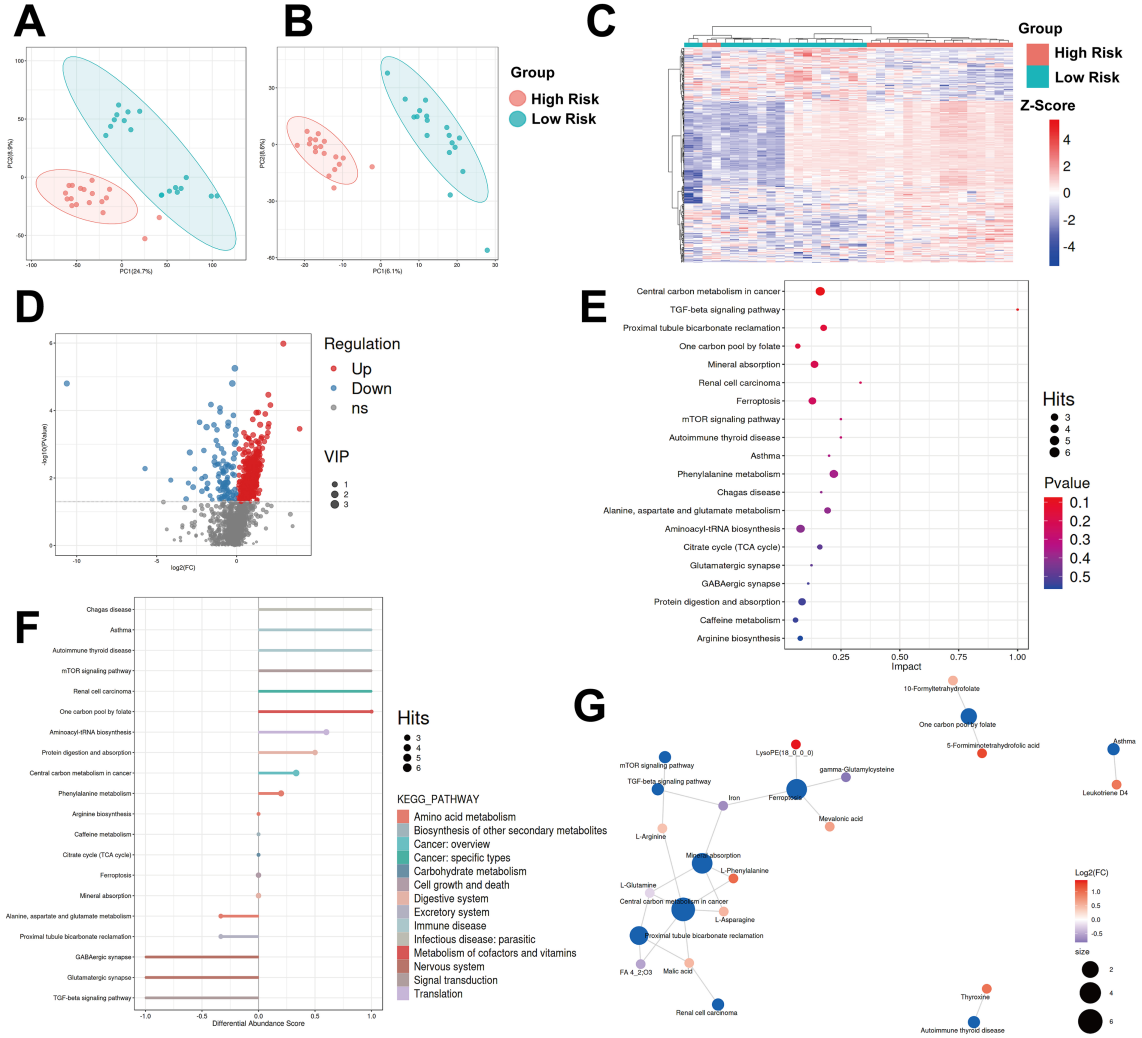
Metabolomics analysis of patients between high- and low combined-score groups. **(A,B)** Principal component analysis scatter plots. The horizontal coordinate PC1 and vertical coordinate PC2, respectively, represent the scores of the first and second principal components. Each scatter represents a sample, and the color and shape of the scatter represent the 95% confidence intervals (Hoteling’s T-squared ellipse) of different grouped samples. **(C)** Heatmap of 1793 differential metabolites. Colored boxes represent upregulation (red) and downregulation (blue) in the high-radiomics-score group. The color scale (upper-right) indicates the fold-change in metabolites expression for all samples. **(D)** Volcano plot of the differential metabolites showing the *p*-value (*y*-axis) and fold-change (*x*-axis) of the quantified metabolites identified in the metabolites in the metabolomic analysis. The metabolites that showed significant downregulation and upregulation are reported in blue and red, respectively. **(E)** Bubble plot of impact factors of top 20 differential metabolic pathways. The *x*-axis represents the impact values of enriched metabolic pathways, and the *y*-axis represents the enriched pathways. The size of the dots indicates the number of metabolites enriched in the pathways. The color is related to the *p*-value. The redder the color, the smaller the *p*-value, and the bluer the color, the larger the *p*-value. **(F)** Differential abundance scores of top 20 differential metabolic pathways. The *x*-axis represents the DA-core value, and the formula is DA-score = (the number of up-regulated substances - the number of down-regulated substances)/the total number of differentially expressed substances in this pathway. The *y*-axis represents the metabolic pathway, and the size of the dots at the top of the columns indicates the number of differentially enriched metabolites in this pathway. **(G)** Top 10 differential metabolic pathways and their associated metabolites. The blue dots represent the pathways, and the other dots represent the metabolites. The size of the pathway dots indicates the number of metabolites connected to them. The more metabolites connected, the larger the dot. The color of the metabolite dots represents the magnitude of the log2(FC) value. Red indicates up-regulation of the difference, and blue indicates down-regulation of the difference. The darker the color, the greater the degree of difference.

Metabolomic analysis was performed on serum samples at admission from 36 patients whose clinical outcome (development of PNAHS or not) was correctly predicted by the combined model. Principal component analysis revealed distinct clustering between the correctly predicted high-risk and low-risk groups, and a total of 1,793 differential metabolites were initially identified (Figures 4A–C). After false discovery rate (FDR) correction (*p* < 0.05), 448 metabolites remained significantly differentially expressed (Figure 4D). Enrichment analysis indicated that these metabolites were primarily involved in pathways related to carbon and amino acid metabolism, and kidney function (Figures 4E,F). Of the eight differentially enriched metabolic pathways containing more than three annotated metabolites, six exhibited a differential abundance scores of at least 0.25, of which four were up-regulated and two were down-regulated. Specifically, several pathways associated with amino acid metabolism and carbon metabolism, as well as ferroptosis, showed significantly different enrichment between the high- and low risk groups. Seven of the top ten enriched pathways formed an interconnected network, mainly linked through amino acids or related intermediates, including L-phenylalanine, L-glutamine, L-arginine, L-asparagine, as well as iron (Figure 4G).

**Figure 5:**
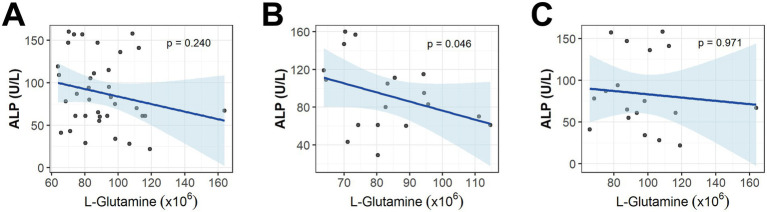
Association analysis between L-glutamine level, clinical indicators, and radiomics features. **(A–C)** Correspond to the regression curves for the overall cohort, the high-risk group, and the low-risk group, respectively. Comparisons were conducted between the value of L-glutamine and that of each clinical or radiomics variable using multi-variable linear regression. Regression coefficient (beta) was adjusted for sex, age, BMI, medical history (hypertension, diabetes, and thrombosis), and GLIM nutritional status. Beta was considered statistically significant at *p* < 0.05.

Given that L-glutamine was the sole downregulated amino acid within this network, considering that glutamine supplementation is frequently incorporated into intestinal rehabilitation regimens, we further investigated the associations between serum L-glutamine levels and baseline clinical and radiomics parameters. Correlation analysis conducted separately across the entire cohort, the high-risk group, and the low-risk group revealed that L-glutamine levels were not significantly associated with most clinical and imaging indicators at baseline (Supplementary Table S8). Notably, however, within the high-risk group, L-glutamine level demonstrated a significant inverse correlation with serum ALP level [beta = −1.42*10^(−8), *p* = 0.046], suggesting that a lower baseline L-glutamine level may be linked to a higher ALP level in these patients. This specific correlation was not evident at the overall population level (Figure 5).

## 4 Discussion

### 4.1 Key findings

In this study, we developed and validated a combined model integrating CT radiomics and clinical features to assess the risk of PNAHS. To our knowledge, this represents the first application of radiomics and deep learning techniques to PNAHS risk prediction. Our findings indicated that the combined radiomics-clinical model demonstrated superior predictive performance compared to models using deep-learning techniques, radiomics or clinical characteristics alone.

### 4.2 Comparison with similar research

While previous studies have successfully employed radiomics and clinical features to predict NAFLD (8, 9, 25–27), this study extends this approach to the distinct context of PNAHS. We demonstrate that the incorporation of radiomics features from non-contrast CT scans, combined with clinical parameters, including TC level, urea level, PN frequency and the L3-intramuscular fat area, constitutes an effective strategy. This finding aligns with the broader paradigm in hepatology research, where multi-parametric models frequently outperform single-modality approaches (28–32). Notably, the specific clinical parameters integrated in our model differ from those highlighted in prior NAFLD radiomics studies, which often included BMI, globulin, ALT, AST, albumin, and triglycerides (7, 9, 29). This discrepancy may stem from fundamental differences in study populations: prior NAFLD cohorts typically comprised patients with established NASH or cirrhosis, whereas our cohort consisted of patients with CIF prior to the onset of PNAHS.

This distinction underscores critical clinical divergence. In NAFLD, reducing dietary fat intake is a cornerstone therapeutic strategy to slow disease progression. However, for patients with CIF, PN-a key risk factor for PNAHS-remains indispensable for short-term nutritional support and health maintenance. Consequently, once PNAHS develops, patients will face the difficult choice of reducing nutritional intake to alleviate the hepatic burden, a strategy that is particularly challenging for patients in poor overall health and unsustainable in the long term. In this context, prevention of PNAHS assumed paramount importance over treatment.

Current diagnostic practices for PNAHS rely primarily on imaging such as ultrasound and CT, as well as hematological markers, with biopsy not recommended as a routine tool (5). Motivated by the imperative for early risk assessment, we conducted this study to establish a predictive model for PNAHS risk, rather than a diagnostic model for established disease.

### 4.3 Explanation about the predictive models

We evaluated the performance of various deep learning architectures against the combined radiomics-clinical model, the clinical model and the traditional radiomics model. The deep learning models encompassed key designs in medical image analysis: classical CNNs (DenseNet121 and ResNet18), Segmentation networks (Unet, SwinUNETR—the latter leveraging Transformers for multi-scale modeling) (33), a VIT for global context, and a hybrid model (Resnet + XGBoost/FC) for feature fusion, alongside baseline MLP and CNN models. However, none of the deep learning models matched the performance of the combined radiomics-clinical model in the testing set.

This may be attributed to the limited sample size constraining the training efficacy of deep learning approached (15). Severe overfitting with small sample datasets remains a major challenge, as evidenced by the marked contrast between the outstanding performance of the deep learning models in the training set and during internal 5-fold cross-validation versus their mediocre generalization to the testing set. Consequently, deep learning may be less suitable for research on certain rare conditions like PNAHS, whereas integrating clinical variables with manually curated radiomics features—allowing guided selection of regions of interest and interpretable feature extraction—can achieve more robust and generalizable performance.

Although the radiomics model was the only one not significantly outperformed by the combined model in testing, its predictive performance was still less satisfactory. This observation, while possibly influenced by the limited testing set size, underscores the superior stability of the combined model. Prior studies in HCC with larger testing sets have shown that both radiomics and combined models can effectively predict prognosis, but similar rigorous validation in benign liver diseases such as NAFLD and PNAHS has been scarce before this investigation (28, 34).

### 4.4 Interpretation of the results from metabolomic analysis

A recent study proposed that amino acids, rather than carbohydrates or fats, serve as the primary carbon source for the tricarboxylic acid (TCA) cycle and lipogenesis in mouse hepatocytes, identifying glutamine as the major contributor (35). In that model, glutamine is initially converted to glutamate and then to metabolites such as *α*-ketoglutarate for entry into the TCA cycle. This is consistent with our finding, in which L-glutamine was identified as the only amino acid exhibiting downregulation that served as a common node connecting multiple top-enriched metabolic pathways. Malic acid, a TCA cycle substrate, was significantly upregulated and directly or indirectly connected to the amino acid metabolism. Although the TCA cycle itself was among the top 20 enriched pathways, no coordinated variation with other pathways was observed in our study. This may be attributed to limited sample size and warrants further investigation.

Ferroptosis is an iron-dependent form of non-apoptotic cell death characterized by excessive lipid peroxidation and is implicated in a wide range of hepatic pathologies. Growing evidence in recent years supports the notion that dysregulation of metabolic pathways and disruption of iron homeostasis contribute to the progression of liver diseases via ferroptosis (35). The complex relationship between ferroptosis and glutamine dependency is a current research focus. Multiple cancer cell lines have been found to resist ferroptosis through the reductive carboxylation of glutamine, while induced glutamine dependency may lead to vulnerability to ferroptosis (36, 37). An animal study have shown that glutamine overexpression in liver metabolic disorders can trigger autophagy-dependent ferroptosis and apoptosis (38). In this study, the ferroptosis pathway was linked to the TGF-beta signaling pathway and showed some association with glutamine-related metabolic pathways. Whether glutamine intake should be restricted in high-risk populations for liver disease, such as patients with CIF, represents a potential direction for future exploration.

Pathways associated with kidney function, including renal cell carcinoma and proximal tube bicarbonate reclamation, were also important parts of the metabolic network in this study. The liver is the primary organ responsible for amino acid metabolism and the urea cycle. Ammonia generated during amino acid catabolism is further converted into urea in the liver. A recent study pointed out that mice with arginine metabolism disorders would have an impaired urea cycle and increased utilization of glutamine at the same time, while the levels of citric acid, malic acid, and fumaric acid were significantly increased, and the risk of developing MASLD was elevated (39). This could help explain the up regulation of L-arginine and increased PNAHS risk contributed by urea level observed in this study, although further exploration is undoubtedly needed.

### 4.5 Clinical relevance

This study provides novel insights into the prediction of PNAHS development in patients with CIF. Our findings suggests that metabolic disturbances capable of predisposing the liver to injury during subsequent nutritional therapy may already be present in some patients, even in the absence of detectable abnormalities in serum liver enzymes or conventional imaging during the initial phase of PN treatment.

The superior performance of the combined model highlights its translational potential for the early identification and stratified management of patients at high PNAHS risk. Implementing such a tool could facilitate timely interventions, potentially improving clinical outcomes and reducing the incidence of severe hepatic complications.

Currently, whether glutamine should be included as part of the intestinal rehabilitation regimen for patients with CIF remains a subject of debate, although some previous studies suggested that it benefits small intestines (1, 2, 40). While the metabolomic profiling in our study identified differential glutamine levels between patients at high versus low risk for PNAHS, further animal experiments and clinical investigation are needed.

### 4.6 Limitations

This study had several limitations. First, as a single-center retrospective study, it is susceptible to selection bias. Although the sample size was sufficient for initial model development, it limits the generalizability of our findings, particularly for clinical outcome prediction, where only 18 patients contributed to analysis in the testing set. Future multi-center studies with larger, more diverse cohorts, including different ethnic groups and age groups (especially pediatric patients, in whom the incidence of CIF has been rising and the mortality is associated with their liver function (41, 42)) are warranted.

Second, experimental evidence supporting the biological interpretation of this study is lacking. The underlying molecular mechanisms and causal relationships between glutamine metabolism and PNAHS risk require further elucidation. Additionally, while non-contrast CT is widely available, variability in scanner models, acquisition parameters, and imaging protocols across institutions and over time may affect the reproducibility of radiomics features, despite the image resampling and normalization applied. Therefore, it is necessary to explore the application of radiomics in other imaging modalities, such as MRI or ultrasound.

## 5 Conclusion

In conclusion, we developed the first predictive model integrating clinical indicators and CT radiomics features to assess the risk of PNAHS in patients with CIF. The distinct risk groups identified by this model exhibited significantly different metabolic profiles, suggesting an underlying association with altered hepatic metabolic function prior to overt liver injury.

## Data Availability

The raw data supporting the conclusions of this article will be made available by the authors, without undue reservation.

## Footnotes

1
https://colab.google

2
https://github.com/ruochenli99/liver_nutrition

## Supplementary material

Supplementary Figure S1 The distribution of selected radiomics features. **(A)** Density plot of selected radiomics features among patients in different sets and with different labels. **(B)** Boxplot of selected radiomics features among patients in different sets and with different labels. The distribution of all the four features was significantly different in the training set. Although the differences of Maximum 2D Diameter Slice and Size Zone NonUniformity Normalized between the two labels were not significant, potential gaps were observed.

Supplementary Figure S2 Clinical application of the combined model. **(A)** Forest plot of the combined model based on characteristics identified as significant via multivariable analysis of the training set (*n* = 219). Significant predictors (*p* < 0.05) from logistic regression are shown. Odds ratios (ORs) are presented, with 95% CIs in parentheses. **(B)** Nomo plot of the combined model. Instruction: Find each of the patient’s values on the according numeric axis (e.g. <=5.18 mmol/L or >5.18 mmol/L for Total Cholesterol), sum all points and find the corresponding risk probability on the bottom axis.
