# Supplementary figures and images for: CT-based radiomics-clinical model for risk assessment of parenteral nutrition-associated hepatic steatosis in chronic intestinal failure and its metabolomic interpretation
Source: Front Nutr. 2026 Feb 3;13:1705520. doi: 10.3389/fnut.2026.1705520 (PMC12909248; doi:10.3389/fnut.2026.1705520)

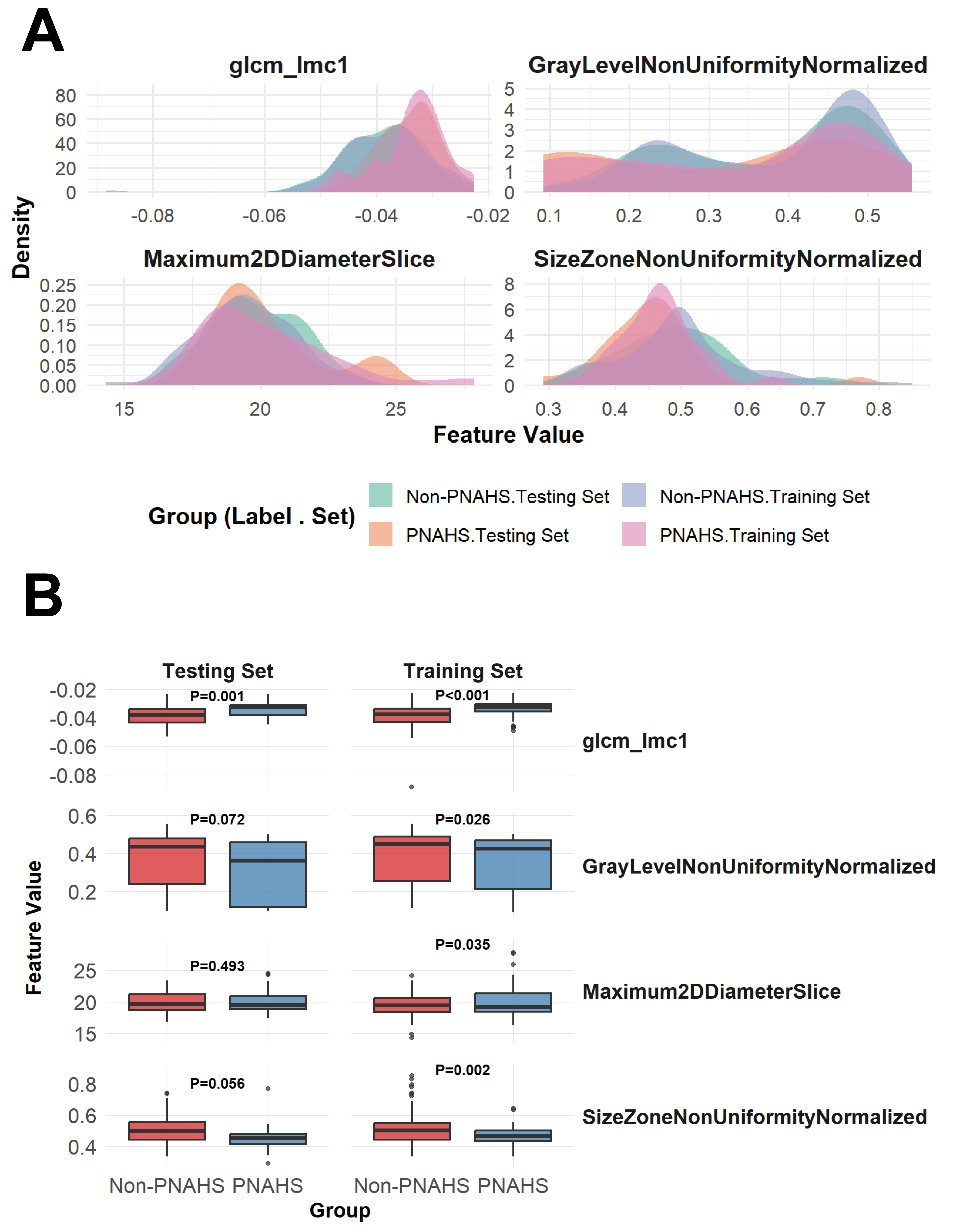

Supplement: Supplementary Figure S1 — The distribution of selected radiomics features. (A) Density plot of selected radiomics features among patients in different sets and with different labels. (B) Boxplot of selected radiomics features among patients in different sets and with different labels. The distribution of all the four features was significantly different in the training set. Although the differences of Maximum 2D Diameter Slice and Size Zone NonUniformity Normalized between the two labels were not significant, potential gaps were observed. [file Image_1.jpeg]

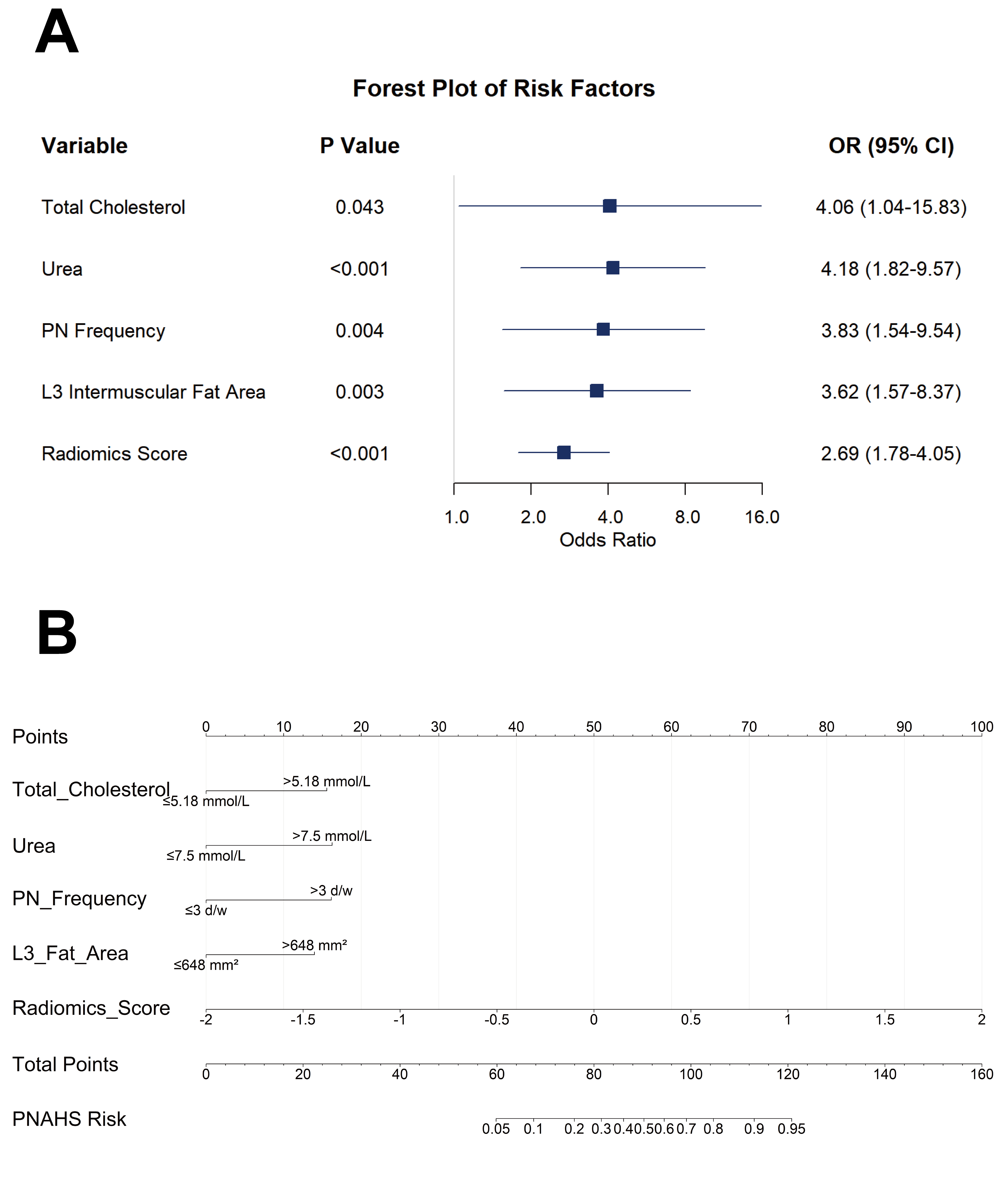

Supplement: Supplementary Figure S2 — Clinical application of the combined model. (A) Forest plot of the combined model based on characteristics identified as significant via multivariable analysis of the training set (n = 219). Significant predictors (p < 0.05) from logistic regression are shown. Odds ratios (ORs) are presented, with 95% CIs in parentheses. (B) Nomo plot of the combined model. Instruction: Find each of the patient’s values on the according numeric axis (e.g. <=5.18 mmol/L or >5.18 mmol/L for Total Cholesterol), sum all points and find the corresponding risk probability on the bottom axis. [file Image_2.jpeg]
