# Supplementary material for: CT-based radiomics-clinical model for risk assessment of parenteral nutrition-associated hepatic steatosis in chronic intestinal failure and its metabolomic interpretation
Source: Front Nutr. 2026 Feb 3;13:1705520. doi: 10.3389/fnut.2026.1705520 (PMC12909248; doi:10.3389/fnut.2026.1705520)
Supplement: Supplementary file 3 [file Table_1.docx]

**CT-based Radiomics-Clinical Model for Risk Assessment of Parenteral Nutrition-associated Hepatic Steatosis in Chronic Intestinal Failure and Its Metabolomic Interpretation**

Yufei Xia^1,2^, Ruochen Li^3^, Sirui Liu^1^, Pinwen Zhou^1^, Jiaqi Wang^1^, Xin Qi^1^, Minyi Zhu^1^, Guangming Sun^1^, Xuejin Gao^1^, Li Zhang^1^, Gulisudumu Maitiabula^1^, Xinying Wang^1,2^*

^1^ Department of General Surgery, Nanjing Jinling Hospital, Affiliated Hospital of Medical School, Nanjing University, Nanjing 210000, China;

^2^ Medical School of Southeast University, Nanjing 210009, China;

^3^ School of Computation, Information and Technology, Technical University of Munich, Munich 80333, Germany.

*Corresponding author(s).
E-mail: wangxinying@nju.edu.cn

**Appendix S1 Radiomics model building processes**

**S1.1 CT Study Protocols**

In this study, abdominal non-contrast CT scans were performed on all included patients using one of the following three CT scanners at the center: Siemens Somatom Definition (Germany), Siemens Definition Flash (Germany), and NeuViz 128 (China). The CT parameters were a tube voltage of 100 kVp or 120 kV, a tube current of 100-700 mA, and a slice thickness of 1.0-6.0 mm.

**S1.2 Preprocessing and feature extraction**

The volumes of interest (VOIs) were segmented by two trained clinicians using ITK-SNAP software (version 3.8.0, University of Pennsylvania and the University of Utah). Of the 307 patients included in the study, CT images were acquired across different time periods: 43 (14.0%) from 2010 and 2014, 110 (35.8%) from 2015 to 2019, and 154 (50.2%) from 2020 to 2023. To mitigate the impact of variations in scanning equipment, protocols and thickness of slices on image quality and, all segmentations were resampled to a voxel size of 1 mm ×1 mm ×1 mm, and voxel intensities were discretized into a range of 25 bins. This preprocessing step ensured that all analyses were conducted based on voxels of identical size and density. After that, 1037 features of 7 types [first order, shape-based, gray level co-occurrence matrix (GLCM), gray level run length matrix (GLRLM), gray level size zone matrix (GLSZM), gray level dependence matrix (GLDM), and neighborhood gray tone difference matrix (NGTDM)] were extracted for each case. The intraclass correlation coefficient (ICC) of all the extracted features from different researchers were calculated to assess stability. Among them, 792 features with an ICC >0.75 were used for subsequent analysis (**Table S1**).

**Table S1. Interclass Correlation Coefficients (ICC) of Radiomic Features**

| **Image Type** | **Feature Type** | **Feature Name** | **ICC (95% CI)** |
| --- | --- | --- | --- |
| **Original** | **First Order** | 10percentile | 1.000 [1.000-1.000] |
|  |  | 90percentile | 1.000 [1.000-1.000] |
|  |  | Energy | 0.997 [0.994-0.999] |
|  |  | Entropy | 0.960 [0.912-0.982] |
|  |  | Interquartile Range | 0.966 [0.925-0.985] |
|  |  | Kurtosis | 0.947 [0.885-0.976] |
|  |  | Maximum | 0.998 [0.995-0.999] |
|  |  | Mean | 1.000 [1.000-1.000] |
|  |  | Mean Absolute Deviation | 0.962 [0.915-0.983] |
|  |  | Median | 1.000 [1.000-1.000] |
|  |  | Minimum | 0.999 [0.998-1.000] |
|  |  | Range | 0.928 [0.843-0.968] |
|  |  | Robust Mean Absolute Deviation | 0.963 [0.919-0.984] |
|  |  | Root Mean Squared | 1.000 [1.000-1.000] |
|  |  | Skewness | 0.754 [0.520-0.883] |
|  |  | Total Energy | 0.997 [0.994-0.999] |
|  |  | Uniformity | 0.962 [0.915-0.983] |
|  |  | Variance | 0.958 [0.908-0.981] |
| **Original** | **GLCM** | Autocorrelation | 0.849 [0.688-0.931] |
|  |  | Cluster Prominence | 0.970 [0.934-0.987] |
|  |  | Cluster Tendency | 0.960 [0.911-0.982] |
|  |  | Contrast | 0.945 [0.879-0.975] |
|  |  | Correlation | 0.908 [0.803-0.959] |
|  |  | Difference Average | 0.951 [0.891-0.978] |
|  |  | Difference Entropy | 0.954 [0.899-0.980] |
|  |  | Difference Variance | 0.944 [0.877-0.975] |
|  |  | Id | 0.956 [0.903-0.980] |
|  |  | Idm | 0.956 [0.903-0.980] |
|  |  | Imc1 | 0.965 [0.921-0.985] |
|  |  | Imc2 | 0.958 [0.907-0.981] |
|  |  | Inverse Variance | 0.940 [0.870-0.973] |
|  |  | Joint Average | 0.848 [0.687-0.930] |
|  |  | Joint Energy | 0.964 [0.920-0.984] |
|  |  | Joint Entropy | 0.956 [0.903-0.980] |
|  |  | Maximum Probability | 0.965 [0.922-0.984] |
|  |  | Mcc | 0.927 [0.843-0.967] |
|  |  | Sum Average | 0.848 [0.687-0.930] |
|  |  | Sum Entropy | 0.954 [0.899-0.979] |
|  |  | Sum Squares | 0.955 [0.901-0.980] |
| **Original** | **GLDM** | Dependence Entropy | 0.954 [0.900-0.979] |
|  |  | Dependence Non Uniformity | 0.977 [0.950-0.990] |
|  |  | Dependence Non Uniformity Normalized | 0.918 [0.823-0.963] |
|  |  | Dependence Variance | 0.919 [0.826-0.964] |
|  |  | Gray Level Non Uniformity | 0.986 [0.968-0.994] |
|  |  | Gray Level Variance | 0.959 [0.909-0.982] |
|  |  | High Gray Level Emphasis | 0.854 [0.697-0.933] |
|  |  | Large Dependence Emphasis | 0.956 [0.903-0.980] |
|  |  | Large Dependence High Gray Level Emphasis | 0.796 [0.591-0.904] |
|  |  | Large Dependence Low Gray Level Emphasis | 0.942 [0.874-0.974] |
|  |  | Low Gray Level Emphasis | 0.832 [0.658-0.922] |
|  |  | Small Dependence Emphasis | 0.915 [0.818-0.961] |
|  |  | Small Dependence High Gray Level Emphasis | 0.857 [0.704-0.934] |
| **Original** | **GLRLM** | Gray Level Non Uniformity | 0.990 [0.977-0.995] |
|  |  | Gray Level Non Uniformity Normalized | 0.958 [0.908-0.981] |
|  |  | Gray Level Variance | 0.958 [0.908-0.981] |
|  |  | High Gray Level Run Emphasis | 0.854 [0.697-0.933] |
|  |  | Long Run Emphasis | 0.961 [0.914-0.983] |
|  |  | Long Run High Gray Level Emphasis | 0.826 [0.645-0.920] |
|  |  | Long Run Low Gray Level Emphasis | 0.905 [0.799-0.957] |
|  |  | Low Gray Level Run Emphasis | 0.828 [0.650-0.920] |
|  |  | Run Entropy | 0.961 [0.914-0.982] |
|  |  | Run Length Non Uniformity | 0.987 [0.972-0.994] |
|  |  | Run Length Non Uniformity Normalized | 0.948 [0.885-0.977] |
|  |  | Run Percentage | 0.955 [0.900-0.980] |
|  |  | Run Variance | 0.963 [0.918-0.984] |
|  |  | Short Run Emphasis | 0.949 [0.887-0.977] |
|  |  | Short Run High Gray Level Emphasis | 0.859 [0.707-0.936] |
|  |  | Short Run Low Gray Level Emphasis | 0.798 [0.596-0.905] |
| **Original** | **GLSZM** | Gray Level Non Uniformity | 0.929 [0.847-0.968] |
|  |  | Gray Level Non Uniformity Normalized | 0.891 [0.769-0.950] |
|  |  | Gray Level Variance | 0.943 [0.875-0.974] |
|  |  | High Gray Level Zone Emphasis | 0.858 [0.706-0.934] |
|  |  | Large Area Emphasis | 0.973 [0.939-0.988] |
|  |  | Large Area High Gray Level Emphasis | 0.843 [0.679-0.927] |
|  |  | Large Area Low Gray Level Emphasis | 0.946 [0.882-0.976] |
|  |  | Size Zone Non Uniformity | 0.943 [0.876-0.974] |
|  |  | Small Area High Gray Level Emphasis | 0.789 [0.581-0.900] |
|  |  | Zone Entropy | 0.894 [0.763-0.953] |
|  |  | Zone Percentage | 0.871 [0.732-0.941] |
|  |  | Zone Variance | 0.974 [0.942-0.988] |
| **Original** | **NGTDM** | Busyness | 0.885 [0.752-0.948] |
|  |  | Coarseness | 0.993 [0.984-0.997] |
|  |  | Complexity | 0.903 [0.792-0.956] |
|  |  | Strength | 0.938 [0.863-0.972] |
| **Original** | **Shape-based** | Flatness | 0.979 [0.952-0.991] |
|  |  | Least Axis Length | 0.975 [0.943-0.989] |
|  |  | Major Axis Length | 0.997 [0.993-0.999] |
|  |  | Maximum2ddiameter Column | 0.971 [0.935-0.987] |
|  |  | Maximum2ddiameter Row | 0.941 [0.872-0.973] |
|  |  | Maximum2ddiameter Slice | 0.994 [0.988-0.998] |
|  |  | Maximum3ddiameter | 0.994 [0.987-0.998] |
|  |  | Mesh Volume | 0.992 [0.981-0.997] |
|  |  | Minor Axis Length | 0.998 [0.995-0.999] |
|  |  | Sphericity | 0.872 [0.734-0.941] |
|  |  | Surface Area | 0.992 [0.982-0.996] |
|  |  | Surface Volume Ratio | 0.981 [0.953-0.992] |
|  |  | Voxel Volume | 0.992 [0.981-0.997] |
| **Square** | **First Order** | Kurtosis | 0.928 [0.845-0.968] |
|  |  | Skewness | 0.754 [0.519-0.883] |
|  |  | Uniformity | 0.758 [0.526-0.885] |
| **Square** | **GLCM** | Autocorrelation | 0.760 [0.533-0.886] |
|  |  | Cluster Prominence | 0.792 [0.584-0.902] |
|  |  | Cluster Shade | 0.893 [0.776-0.951] |
|  |  | Correlation | 0.891 [0.767-0.950] |
|  |  | Imc1 | 0.965 [0.923-0.985] |
|  |  | Imc2 | 0.891 [0.768-0.950] |
|  |  | Joint Energy | 0.790 [0.581-0.901] |
|  |  | Maximum Probability | 0.787 [0.577-0.900] |
|  |  | Mcc | 0.907 [0.802-0.958] |
| **Square** | **GLDM** | Dependence Entropy | 0.797 [0.592-0.906] |
|  |  | Dependence Non Uniformity | 0.885 [0.756-0.948] |
|  |  | Gray Level Non Uniformity | 0.848 [0.688-0.930] |
|  |  | High Gray Level Emphasis | 0.756 [0.526-0.884] |
|  |  | Large Dependence Emphasis | 0.764 [0.536-0.888] |
|  |  | Large Dependence High Gray Level Emphasis | 0.849 [0.691-0.930] |
|  |  | Low Gray Level Emphasis | 0.756 [0.526-0.884] |
| **Square** | **GLRLM** | Gray Level Non Uniformity | 0.864 [0.718-0.937] |
|  |  | Gray Level Non Uniformity Normalized | 0.751 [0.514-0.882] |
|  |  | High Gray Level Run Emphasis | 0.757 [0.528-0.885] |
|  |  | Long Run Emphasis | 0.781 [0.565-0.897] |
|  |  | Long Run High Gray Level Emphasis | 0.789 [0.582-0.901] |
|  |  | Low Gray Level Run Emphasis | 0.750 [0.516-0.881] |
|  |  | Run Length Non Uniformity | 0.943 [0.874-0.974] |
|  |  | Run Variance | 0.800 [0.600-0.907] |
|  |  | Short Run Low Gray Level Emphasis | 0.758 [0.529-0.885] |
| **Square** | **GLSZM** | Gray Level Non Uniformity | 0.941 [0.872-0.974] |
|  |  | Gray Level Non Uniformity Normalized | 0.812 [0.620-0.912] |
|  |  | High Gray Level Zone Emphasis | 0.779 [0.564-0.895] |
|  |  | Large Area Emphasis | 0.846 [0.684-0.929] |
|  |  | Large Area Low Gray Level Emphasis | 0.879 [0.747-0.944] |
|  |  | Size Zone Non Uniformity | 0.761 [0.531-0.887] |
|  |  | Small Area High Gray Level Emphasis | 0.780 [0.566-0.896] |
|  |  | Zone Variance | 0.847 [0.687-0.929] |
| **Square** | **NGTDM** | Busyness | 0.848 [0.689-0.930] |
|  |  | Coarseness | 0.993 [0.985-0.997] |
|  |  | Strength | 0.915 [0.819-0.962] |
| **Square Root** | **First Order** | 10percentile | 0.854 [0.699-0.933] |
|  |  | 90percentile | 0.847 [0.686-0.930] |
|  |  | Energy | 0.857 [0.706-0.934] |
|  |  | Entropy | 0.838 [0.669-0.925] |
|  |  | Interquartile Range | 0.841 [0.674-0.927] |
|  |  | Kurtosis | 0.953 [0.897-0.979] |
|  |  | Maximum | 0.842 [0.677-0.927] |
|  |  | Mean | 0.851 [0.692-0.931] |
|  |  | Mean Absolute Deviation | 0.829 [0.653-0.920] |
|  |  | Median | 0.851 [0.692-0.931] |
|  |  | Minimum | 0.863 [0.716-0.937] |
|  |  | Range | 0.814 [0.622-0.914] |
|  |  | Robust Mean Absolute Deviation | 0.828 [0.652-0.920] |
|  |  | Root Mean Squared | 0.851 [0.692-0.931] |
|  |  | Skewness | 0.773 [0.552-0.893] |
|  |  | Total Energy | 0.857 [0.706-0.934] |
|  |  | Uniformity | 0.876 [0.742-0.943] |
|  |  | Variance | 0.836 [0.667-0.924] |
| **Square Root** | **GLCM** | Autocorrelation | 0.789 [0.575-0.901] |
|  |  | Cluster Prominence | 0.928 [0.844-0.967] |
|  |  | Cluster Tendency | 0.882 [0.753-0.946] |
|  |  | Correlation | 0.912 [0.811-0.960] |
|  |  | Difference Average | 0.787 [0.578-0.900] |
|  |  | Difference Entropy | 0.797 [0.595-0.905] |
|  |  | Difference Variance | 0.759 [0.529-0.885] |
|  |  | Id | 0.821 [0.638-0.916] |
|  |  | Idm | 0.810 [0.618-0.911] |
|  |  | Imc1 | 0.956 [0.901-0.981] |
|  |  | Imc2 | 0.942 [0.865-0.975] |
|  |  | Inverse Variance | 0.918 [0.824-0.963] |
|  |  | Joint Average | 0.768 [0.539-0.891] |
|  |  | Joint Energy | 0.908 [0.806-0.958] |
|  |  | Joint Entropy | 0.836 [0.666-0.924] |
|  |  | Maximum Probability | 0.775 [0.558-0.894] |
|  |  | Mcc | 0.904 [0.796-0.957] |
|  |  | Sum Average | 0.768 [0.539-0.891] |
|  |  | Sum Entropy | 0.867 [0.724-0.939] |
|  |  | Sum Squares | 0.835 [0.665-0.923] |
| **Square Root** | **GLDM** | Dependence Entropy | 0.917 [0.817-0.963] |
|  |  | Dependence Non Uniformity | 0.930 [0.849-0.969] |
|  |  | Dependence Variance | 0.775 [0.554-0.894] |
|  |  | Gray Level Non Uniformity | 0.965 [0.923-0.984] |
|  |  | Gray Level Variance | 0.831 [0.658-0.922] |
|  |  | High Gray Level Emphasis | 0.791 [0.580-0.903] |
|  |  | Large Dependence Emphasis | 0.870 [0.730-0.940] |
|  |  | Large Dependence High Gray Level Emphasis | 0.759 [0.529-0.886] |
|  |  | Large Dependence Low Gray Level Emphasis | 0.787 [0.572-0.900] |
| **Square Root** | **GLRLM** | Gray Level Non Uniformity | 0.987 [0.972-0.994] |
|  |  | Gray Level Non Uniformity Normalized | 0.782 [0.570-0.897] |
|  |  | Gray Level Variance | 0.817 [0.631-0.914] |
|  |  | High Gray Level Run Emphasis | 0.792 [0.582-0.903] |
|  |  | Long Run Emphasis | 0.937 [0.864-0.972] |
|  |  | Long Run High Gray Level Emphasis | 0.810 [0.616-0.911] |
|  |  | Long Run Low Gray Level Emphasis | 0.815 [0.623-0.914] |
|  |  | Run Entropy | 0.902 [0.786-0.956] |
|  |  | Run Length Non Uniformity | 0.935 [0.859-0.971] |
|  |  | Run Length Non Uniformity Normalized | 0.784 [0.572-0.898] |
|  |  | Run Percentage | 0.838 [0.669-0.925] |
|  |  | Run Variance | 0.942 [0.875-0.974] |
|  |  | Short Run Emphasis | 0.797 [0.595-0.905] |
|  |  | Short Run High Gray Level Emphasis | 0.780 [0.560-0.897] |
| **Square Root** | **GLSZM** | Gray Level Non Uniformity | 0.835 [0.663-0.924] |
|  |  | Gray Level Variance | 0.793 [0.584-0.903] |
|  |  | Size Zone Non Uniformity | 0.809 [0.612-0.911] |
| **Square Root** | **NGTDM** | Coarseness | 0.925 [0.838-0.966] |
|  |  | Strength | 0.894 [0.775-0.952] |
| **Wavelet-HHH** | **First Order** | 10percentile | 0.917 [0.805-0.964] |
|  |  | 90percentile | 0.941 [0.849-0.976] |
|  |  | Energy | 0.977 [0.949-0.990] |
|  |  | Entropy | 0.937 [0.860-0.972] |
|  |  | Interquartile Range | 0.942 [0.842-0.976] |
|  |  | Maximum | 0.753 [0.518-0.883] |
|  |  | Mean Absolute Deviation | 0.944 [0.849-0.977] |
|  |  | Range | 0.772 [0.553-0.892] |
|  |  | Robust Mean Absolute Deviation | 0.942 [0.832-0.977] |
|  |  | Root Mean Squared | 0.944 [0.856-0.977] |
|  |  | Total Energy | 0.977 [0.949-0.990] |
|  |  | Uniformity | 0.932 [0.850-0.970] |
|  |  | Variance | 0.932 [0.830-0.971] |
| **Wavelet-HHH** | **GLCM** | Cluster Prominence | 0.901 [0.781-0.956] |
|  |  | Cluster Tendency | 0.917 [0.815-0.963] |
|  |  | Contrast | 0.916 [0.812-0.963] |
|  |  | Difference Average | 0.920 [0.821-0.965] |
|  |  | Difference Entropy | 0.938 [0.860-0.972] |
|  |  | Difference Variance | 0.925 [0.832-0.967] |
|  |  | Id | 0.922 [0.826-0.965] |
|  |  | Idm | 0.922 [0.826-0.965] |
|  |  | Imc1 | 0.756 [0.514-0.885] |
|  |  | Imc2 | 0.775 [0.540-0.896] |
|  |  | Inverse Variance | 0.837 [0.664-0.925] |
|  |  | Joint Energy | 0.936 [0.857-0.971] |
|  |  | Joint Entropy | 0.934 [0.853-0.971] |
|  |  | Maximum Probability | 0.923 [0.836-0.965] |
|  |  | Mcc | 0.750 [0.495-0.884] |
|  |  | Sum Entropy | 0.936 [0.857-0.972] |
|  |  | Sum Squares | 0.917 [0.814-0.963] |
| **Wavelet-HHH** | **GLDM** | Dependence Entropy | 0.914 [0.812-0.962] |
|  |  | Dependence Non Uniformity | 0.980 [0.950-0.991] |
|  |  | Dependence Non Uniformity Normalized | 0.886 [0.759-0.948] |
|  |  | Dependence Variance | 0.946 [0.882-0.976] |
|  |  | Gray Level Non Uniformity | 0.983 [0.957-0.993] |
|  |  | Gray Level Variance | 0.921 [0.824-0.965] |
|  |  | Large Dependence Emphasis | 0.886 [0.748-0.949] |
|  |  | Small Dependence Emphasis | 0.920 [0.828-0.964] |
| **Wavelet-HHH** | **GLRLM** | Gray Level Non Uniformity | 0.986 [0.966-0.994] |
|  |  | Gray Level Non Uniformity Normalized | 0.943 [0.873-0.974] |
|  |  | Gray Level Variance | 0.929 [0.843-0.969] |
|  |  | Long Run Emphasis | 0.900 [0.779-0.955] |
|  |  | Run Entropy | 0.978 [0.951-0.990] |
|  |  | Run Length Non Uniformity | 0.990 [0.977-0.996] |
|  |  | Run Length Non Uniformity Normalized | 0.903 [0.789-0.956] |
|  |  | Run Percentage | 0.894 [0.765-0.953] |
|  |  | Run Variance | 0.876 [0.726-0.945] |
|  |  | Short Run Emphasis | 0.916 [0.817-0.963] |
| **Wavelet-HHH** | **GLSZM** | Gray Level Non Uniformity | 0.967 [0.928-0.985] |
|  |  | Gray Level Non Uniformity Normalized | 0.809 [0.608-0.912] |
|  |  | Gray Level Variance | 0.834 [0.643-0.925] |
|  |  | Large Area Emphasis | 0.935 [0.859-0.971] |
|  |  | Large Area High Gray Level Emphasis | 0.777 [0.559-0.895] |
|  |  | Large Area Low Gray Level Emphasis | 0.905 [0.799-0.957] |
|  |  | Size Zone Non Uniformity | 0.926 [0.837-0.967] |
|  |  | Size Zone Non Uniformity Normalized | 0.844 [0.680-0.928] |
|  |  | Zone Entropy | 0.903 [0.776-0.957] |
|  |  | Zone Variance | 0.930 [0.849-0.968] |
| **Wavelet-HHH** | **NGTDM** | Coarseness | 0.986 [0.963-0.994] |
| **Wavelet-HHL** | **First Order** | 10percentile | 0.962 [0.917-0.983] |
|  |  | 90percentile | 0.964 [0.920-0.984] |
|  |  | Energy | 0.977 [0.949-0.990] |
|  |  | Entropy | 0.974 [0.942-0.988] |
|  |  | Interquartile Range | 0.954 [0.900-0.980] |
|  |  | Kurtosis | 0.840 [0.673-0.926] |
|  |  | Maximum | 0.952 [0.895-0.978] |
|  |  | Mean Absolute Deviation | 0.967 [0.928-0.985] |
|  |  | Minimum | 0.921 [0.831-0.964] |
|  |  | Range | 0.975 [0.943-0.989] |
|  |  | Robust Mean Absolute Deviation | 0.962 [0.916-0.983] |
|  |  | Root Mean Squared | 0.972 [0.937-0.987] |
|  |  | Total Energy | 0.977 [0.949-0.990] |
|  |  | Uniformity | 0.973 [0.939-0.988] |
|  |  | Variance | 0.967 [0.927-0.985] |
| **Wavelet-HHL** | **GLCM** | Autocorrelation | 0.826 [0.643-0.919] |
|  |  | Cluster Prominence | 0.965 [0.923-0.984] |
|  |  | Cluster Tendency | 0.963 [0.918-0.983] |
|  |  | Contrast | 0.962 [0.916-0.983] |
|  |  | Correlation | 0.853 [0.695-0.932] |
|  |  | Difference Average | 0.965 [0.922-0.984] |
|  |  | Difference Entropy | 0.972 [0.938-0.987] |
|  |  | Difference Variance | 0.968 [0.929-0.986] |
|  |  | Id | 0.968 [0.928-0.986] |
|  |  | Idm | 0.967 [0.927-0.985] |
|  |  | Imc1 | 0.887 [0.761-0.949] |
|  |  | Imc2 | 0.914 [0.814-0.961] |
|  |  | Inverse Variance | 0.932 [0.852-0.970] |
|  |  | Joint Average | 0.817 [0.627-0.915] |
|  |  | Joint Energy | 0.975 [0.944-0.989] |
|  |  | Joint Entropy | 0.972 [0.938-0.987] |
|  |  | Maximum Probability | 0.972 [0.938-0.987] |
|  |  | Mcc | 0.890 [0.766-0.950] |
|  |  | Sum Average | 0.817 [0.627-0.915] |
|  |  | Sum Entropy | 0.972 [0.937-0.987] |
|  |  | Sum Squares | 0.963 [0.917-0.983] |
| **Wavelet-HHL** | **GLDM** | Dependence Entropy | 0.964 [0.921-0.984] |
|  |  | Dependence Non Uniformity | 0.981 [0.957-0.991] |
|  |  | Dependence Non Uniformity Normalized | 0.866 [0.721-0.938] |
|  |  | Dependence Variance | 0.850 [0.692-0.931] |
|  |  | Gray Level Non Uniformity | 0.982 [0.957-0.992] |
|  |  | Gray Level Variance | 0.966 [0.925-0.985] |
|  |  | High Gray Level Emphasis | 0.840 [0.670-0.926] |
|  |  | Large Dependence Emphasis | 0.961 [0.915-0.983] |
|  |  | Large Dependence Low Gray Level Emphasis | 0.800 [0.602-0.906] |
|  |  | Small Dependence Emphasis | 0.956 [0.903-0.980] |
|  |  | Small Dependence High Gray Level Emphasis | 0.898 [0.782-0.954] |
| **Wavelet-HHL** | **GLRLM** | Gray Level Non Uniformity | 0.989 [0.973-0.995] |
|  |  | Gray Level Non Uniformity Normalized | 0.977 [0.948-0.990] |
|  |  | Gray Level Variance | 0.970 [0.932-0.987] |
|  |  | High Gray Level Run Emphasis | 0.841 [0.672-0.927] |
|  |  | Long Run Emphasis | 0.967 [0.928-0.985] |
|  |  | Run Entropy | 0.975 [0.946-0.989] |
|  |  | Run Length Non Uniformity | 0.987 [0.970-0.994] |
|  |  | Run Length Non Uniformity Normalized | 0.967 [0.928-0.985] |
|  |  | Run Percentage | 0.965 [0.923-0.984] |
|  |  | Run Variance | 0.963 [0.919-0.984] |
|  |  | Short Run Emphasis | 0.970 [0.933-0.986] |
|  |  | Short Run High Gray Level Emphasis | 0.867 [0.721-0.939] |
| **Wavelet-HHL** | **GLSZM** | Gray Level Non Uniformity | 0.890 [0.770-0.950] |
|  |  | Gray Level Non Uniformity Normalized | 0.958 [0.906-0.981] |
|  |  | Gray Level Variance | 0.961 [0.913-0.982] |
|  |  | High Gray Level Zone Emphasis | 0.842 [0.676-0.927] |
|  |  | Large Area Emphasis | 0.959 [0.910-0.982] |
|  |  | Large Area High Gray Level Emphasis | 0.875 [0.737-0.943] |
|  |  | Large Area Low Gray Level Emphasis | 0.952 [0.895-0.978] |
|  |  | Size Zone Non Uniformity | 0.965 [0.923-0.984] |
|  |  | Size Zone Non Uniformity Normalized | 0.823 [0.638-0.918] |
|  |  | Small Area Emphasis | 0.798 [0.594-0.906] |
|  |  | Small Area High Gray Level Emphasis | 0.836 [0.663-0.925] |
|  |  | Zone Entropy | 0.960 [0.913-0.982] |
|  |  | Zone Percentage | 0.816 [0.629-0.915] |
|  |  | Zone Variance | 0.961 [0.914-0.983] |
| **Wavelet-HHL** | **NGTDM** | Busyness | 0.807 [0.608-0.910] |
|  |  | Coarseness | 0.989 [0.975-0.995] |
|  |  | Complexity | 0.924 [0.837-0.966] |
|  |  | Strength | 0.867 [0.722-0.939] |
| **Wavelet-Hlh** | **First Order** | 10percentile | 0.930 [0.849-0.968] |
|  |  | 90percentile | 0.947 [0.884-0.976] |
|  |  | Energy | 0.971 [0.936-0.987] |
|  |  | Entropy | 0.938 [0.865-0.972] |
|  |  | Interquartile Range | 0.919 [0.824-0.963] |
|  |  | Maximum | 0.832 [0.655-0.922] |
|  |  | Mean Absolute Deviation | 0.935 [0.859-0.971] |
|  |  | Minimum | 0.896 [0.781-0.953] |
|  |  | Range | 0.902 [0.792-0.955] |
|  |  | Robust Mean Absolute Deviation | 0.925 [0.838-0.966] |
|  |  | Root Mean Squared | 0.938 [0.866-0.972] |
|  |  | Total Energy | 0.971 [0.936-0.987] |
|  |  | Uniformity | 0.937 [0.863-0.972] |
|  |  | Variance | 0.940 [0.870-0.973] |
| **Wavelet-Hlh** | **GLCM** | Autocorrelation | 0.900 [0.787-0.955] |
|  |  | Cluster Prominence | 0.940 [0.868-0.973] |
|  |  | Cluster Tendency | 0.940 [0.868-0.973] |
|  |  | Contrast | 0.942 [0.873-0.974] |
|  |  | Correlation | 0.779 [0.527-0.900] |
|  |  | Difference Average | 0.939 [0.867-0.973] |
|  |  | Difference Entropy | 0.943 [0.876-0.974] |
|  |  | Difference Variance | 0.943 [0.877-0.975] |
|  |  | Id | 0.936 [0.860-0.971] |
|  |  | Idm | 0.936 [0.861-0.971] |
|  |  | Imc1 | 0.843 [0.679-0.927] |
|  |  | Imc2 | 0.870 [0.729-0.940] |
|  |  | Inverse Variance | 0.932 [0.853-0.969] |
|  |  | Joint Average | 0.897 [0.782-0.953] |
|  |  | Joint Energy | 0.942 [0.873-0.974] |
|  |  | Joint Entropy | 0.941 [0.871-0.973] |
|  |  | Maximum Probability | 0.939 [0.868-0.973] |
|  |  | Mcc | 0.818 [0.634-0.915] |
|  |  | Sum Average | 0.897 [0.782-0.953] |
|  |  | Sum Entropy | 0.940 [0.869-0.973] |
|  |  | Sum Squares | 0.941 [0.871-0.973] |
| **Wavelet-Hlh** | **GLDM** | Dependence Entropy | 0.893 [0.773-0.952] |
|  |  | Dependence Non Uniformity | 0.985 [0.962-0.994] |
|  |  | Gray Level Non Uniformity | 0.959 [0.909-0.982] |
|  |  | Gray Level Variance | 0.941 [0.871-0.973] |
|  |  | High Gray Level Emphasis | 0.903 [0.794-0.956] |
|  |  | Large Dependence Emphasis | 0.915 [0.818-0.962] |
|  |  | Large Dependence High Gray Level Emphasis | 0.766 [0.541-0.889] |
|  |  | Large Dependence Low Gray Level Emphasis | 0.956 [0.904-0.981] |
|  |  | Low Gray Level Emphasis | 0.934 [0.858-0.970] |
|  |  | Small Dependence Emphasis | 0.865 [0.720-0.938] |
|  |  | Small Dependence High Gray Level Emphasis | 0.930 [0.848-0.969] |
|  |  | Small Dependence Low Gray Level Emphasis | 0.876 [0.742-0.943] |
| **Wavelet-Hlh** | **GLRLM** | Gray Level Non Uniformity | 0.977 [0.947-0.990] |
|  |  | Gray Level Non Uniformity Normalized | 0.940 [0.870-0.973] |
|  |  | Gray Level Variance | 0.941 [0.872-0.973] |
|  |  | High Gray Level Run Emphasis | 0.904 [0.796-0.957] |
|  |  | Long Run Emphasis | 0.924 [0.838-0.966] |
|  |  | Long Run High Gray Level Emphasis | 0.838 [0.670-0.925] |
|  |  | Long Run Low Gray Level Emphasis | 0.955 [0.901-0.980] |
|  |  | Low Gray Level Run Emphasis | 0.936 [0.861-0.971] |
|  |  | Run Entropy | 0.941 [0.871-0.973] |
|  |  | Run Length Non Uniformity | 0.984 [0.965-0.993] |
|  |  | Run Length Non Uniformity Normalized | 0.920 [0.827-0.964] |
|  |  | Run Percentage | 0.919 [0.825-0.963] |
|  |  | Run Variance | 0.910 [0.809-0.959] |
|  |  | Short Run Emphasis | 0.921 [0.831-0.965] |
|  |  | Short Run High Gray Level Emphasis | 0.916 [0.819-0.962] |
|  |  | Short Run Low Gray Level Emphasis | 0.926 [0.840-0.966] |
| **Wavelet-Hlh** | **GLSZM** | Gray Level Non Uniformity | 0.829 [0.648-0.921] |
|  |  | Gray Level Non Uniformity Normalized | 0.881 [0.749-0.946] |
|  |  | Gray Level Variance | 0.933 [0.855-0.970] |
|  |  | High Gray Level Zone Emphasis | 0.903 [0.792-0.956] |
|  |  | Large Area Emphasis | 0.833 [0.661-0.922] |
|  |  | Large Area High Gray Level Emphasis | 0.899 [0.781-0.954] |
|  |  | Large Area Low Gray Level Emphasis | 0.829 [0.649-0.921] |
|  |  | Low Gray Level Zone Emphasis | 0.934 [0.857-0.970] |
|  |  | Size Zone Non Uniformity | 0.904 [0.795-0.957] |
|  |  | Zone Entropy | 0.920 [0.826-0.964] |
|  |  | Zone Variance | 0.842 [0.677-0.927] |
| **Wavelet-Hlh** | **NGTDM** | Coarseness | 0.987 [0.970-0.994] |
|  |  | Complexity | 0.851 [0.690-0.931] |
| **Wavelet-HLL** | **First Order** | 10percentile | 0.959 [0.910-0.982] |
|  |  | 90percentile | 0.963 [0.918-0.984] |
|  |  | Energy | 0.975 [0.944-0.989] |
|  |  | Entropy | 0.964 [0.919-0.984] |
|  |  | Interquartile Range | 0.966 [0.924-0.985] |
|  |  | Kurtosis | 0.881 [0.749-0.946] |
|  |  | Maximum | 0.914 [0.815-0.961] |
|  |  | Mean Absolute Deviation | 0.966 [0.925-0.985] |
|  |  | Minimum | 0.934 [0.857-0.971] |
|  |  | Range | 0.955 [0.901-0.980] |
|  |  | Robust Mean Absolute Deviation | 0.965 [0.922-0.984] |
|  |  | Root Mean Squared | 0.967 [0.928-0.985] |
|  |  | Total Energy | 0.975 [0.944-0.989] |
|  |  | Uniformity | 0.955 [0.900-0.980] |
|  |  | Variance | 0.968 [0.929-0.986] |
| **Wavelet-HLL** | **GLCM** | Autocorrelation | 0.919 [0.826-0.963] |
|  |  | Cluster Prominence | 0.970 [0.934-0.987] |
|  |  | Cluster Tendency | 0.964 [0.920-0.984] |
|  |  | Contrast | 0.970 [0.933-0.987] |
|  |  | Correlation | 0.960 [0.911-0.982] |
|  |  | Difference Average | 0.966 [0.925-0.985] |
|  |  | Difference Entropy | 0.966 [0.924-0.985] |
|  |  | Difference Variance | 0.971 [0.937-0.987] |
|  |  | Id | 0.959 [0.910-0.982] |
|  |  | Idm | 0.960 [0.910-0.982] |
|  |  | Imc1 | 0.865 [0.721-0.938] |
|  |  | Imc2 | 0.933 [0.854-0.970] |
|  |  | Inverse Variance | 0.962 [0.916-0.983] |
|  |  | Joint Average | 0.922 [0.831-0.965] |
|  |  | Joint Energy | 0.951 [0.893-0.978] |
|  |  | Joint Entropy | 0.962 [0.916-0.983] |
|  |  | Maximum Probability | 0.948 [0.886-0.976] |
|  |  | Mcc | 0.932 [0.853-0.969] |
|  |  | Sum Average | 0.922 [0.831-0.965] |
|  |  | Sum Entropy | 0.960 [0.912-0.982] |
|  |  | Sum Squares | 0.967 [0.927-0.985] |
| **Wavelet-HLL** | **GLDM** | Dependence Entropy | 0.920 [0.828-0.964] |
|  |  | Dependence Non Uniformity | 0.984 [0.963-0.993] |
|  |  | Dependence Non Uniformity Normalized | 0.946 [0.883-0.976] |
|  |  | Dependence Variance | 0.916 [0.822-0.962] |
|  |  | Gray Level Non Uniformity | 0.964 [0.922-0.984] |
|  |  | Gray Level Variance | 0.968 [0.930-0.986] |
|  |  | High Gray Level Emphasis | 0.925 [0.839-0.966] |
|  |  | Large Dependence Emphasis | 0.939 [0.868-0.973] |
|  |  | Large Dependence High Gray Level Emphasis | 0.782 [0.567-0.898] |
|  |  | Large Dependence Low Gray Level Emphasis | 0.963 [0.919-0.984] |
|  |  | Low Gray Level Emphasis | 0.917 [0.820-0.962] |
|  |  | Small Dependence Emphasis | 0.950 [0.891-0.978] |
|  |  | Small Dependence High Gray Level Emphasis | 0.966 [0.926-0.985] |
| **Wavelet-HLL** | **GLRLM** | Gray Level Non Uniformity | 0.980 [0.956-0.991] |
|  |  | Gray Level Non Uniformity Normalized | 0.958 [0.906-0.981] |
|  |  | Gray Level Variance | 0.969 [0.931-0.986] |
|  |  | High Gray Level Run Emphasis | 0.926 [0.841-0.967] |
|  |  | Long Run Emphasis | 0.941 [0.872-0.974] |
|  |  | Long Run High Gray Level Emphasis | 0.889 [0.766-0.950] |
|  |  | Long Run Low Gray Level Emphasis | 0.953 [0.896-0.979] |
|  |  | Low Gray Level Run Emphasis | 0.916 [0.819-0.962] |
|  |  | Run Entropy | 0.971 [0.936-0.987] |
|  |  | Run Length Non Uniformity | 0.986 [0.970-0.994] |
|  |  | Run Length Non Uniformity Normalized | 0.949 [0.889-0.977] |
|  |  | Run Percentage | 0.947 [0.883-0.976] |
|  |  | Run Variance | 0.939 [0.867-0.972] |
|  |  | Short Run Emphasis | 0.948 [0.887-0.977] |
|  |  | Short Run High Gray Level Emphasis | 0.932 [0.853-0.969] |
|  |  | Short Run Low Gray Level Emphasis | 0.898 [0.783-0.954] |
| **Wavelet-HLL** | **GLSZM** | Gray Level Non Uniformity | 0.978 [0.951-0.990] |
|  |  | Gray Level Non Uniformity Normalized | 0.961 [0.915-0.983] |
|  |  | Gray Level Variance | 0.968 [0.929-0.986] |
|  |  | High Gray Level Zone Emphasis | 0.952 [0.896-0.979] |
|  |  | Large Area Emphasis | 0.913 [0.813-0.961] |
|  |  | Large Area Low Gray Level Emphasis | 0.944 [0.877-0.975] |
|  |  | Low Gray Level Zone Emphasis | 0.861 [0.709-0.936] |
|  |  | Size Zone Non Uniformity | 0.962 [0.916-0.983] |
|  |  | Small Area High Gray Level Emphasis | 0.959 [0.909-0.982] |
|  |  | Small Area Low Gray Level Emphasis | 0.786 [0.572-0.900] |
|  |  | Zone Entropy | 0.881 [0.743-0.946] |
|  |  | Zone Percentage | 0.945 [0.880-0.975] |
|  |  | Zone Variance | 0.914 [0.816-0.961] |
| **Wavelet-HLL** | **NGTDM** | Busyness | 0.925 [0.837-0.966] |
|  |  | Coarseness | 0.993 [0.984-0.997] |
|  |  | Complexity | 0.950 [0.891-0.978] |
|  |  | Contrast | 0.823 [0.638-0.918] |
|  |  | Strength | 0.939 [0.867-0.973] |
| **Wavelet-LHH** | **First Order** | 10percentile | 0.938 [0.864-0.972] |
|  |  | 90percentile | 0.940 [0.870-0.973] |
|  |  | Energy | 0.961 [0.914-0.983] |
|  |  | Entropy | 0.951 [0.893-0.978] |
|  |  | Interquartile Range | 0.943 [0.877-0.975] |
|  |  | Maximum | 0.918 [0.823-0.963] |
|  |  | Mean Absolute Deviation | 0.954 [0.899-0.979] |
|  |  | Minimum | 0.753 [0.502-0.885] |
|  |  | Range | 0.923 [0.828-0.966] |
|  |  | Robust Mean Absolute Deviation | 0.955 [0.901-0.980] |
|  |  | Root Mean Squared | 0.951 [0.894-0.978] |
|  |  | Total Energy | 0.961 [0.914-0.983] |
|  |  | Uniformity | 0.952 [0.896-0.979] |
|  |  | Variance | 0.948 [0.887-0.977] |
| **Wavelet-LHH** | **GLCM** | Autocorrelation | 0.757 [0.493-0.889] |
|  |  | Cluster Prominence | 0.948 [0.887-0.977] |
|  |  | Cluster Tendency | 0.949 [0.888-0.977] |
|  |  | Contrast | 0.946 [0.883-0.976] |
|  |  | Difference Average | 0.951 [0.892-0.978] |
|  |  | Difference Entropy | 0.952 [0.896-0.978] |
|  |  | Difference Variance | 0.944 [0.879-0.975] |
|  |  | Id | 0.954 [0.900-0.979] |
|  |  | Idm | 0.954 [0.900-0.979] |
|  |  | Imc1 | 0.895 [0.776-0.952] |
|  |  | Imc2 | 0.875 [0.740-0.943] |
|  |  | Inverse Variance | 0.911 [0.808-0.960] |
|  |  | Joint Energy | 0.961 [0.915-0.983] |
|  |  | Joint Entropy | 0.955 [0.901-0.980] |
|  |  | Maximum Probability | 0.944 [0.878-0.975] |
|  |  | Mcc | 0.886 [0.759-0.948] |
|  |  | Sum Entropy | 0.956 [0.904-0.980] |
|  |  | Sum Squares | 0.948 [0.886-0.977] |
| **Wavelet-LHH** | **GLDM** | Dependence Entropy | 0.908 [0.803-0.958] |
|  |  | Dependence Non Uniformity | 0.989 [0.976-0.995] |
|  |  | Dependence Non Uniformity Normalized | 0.935 [0.860-0.971] |
|  |  | Dependence Variance | 0.942 [0.874-0.974] |
|  |  | Gray Level Non Uniformity | 0.993 [0.981-0.997] |
|  |  | Gray Level Variance | 0.945 [0.881-0.975] |
|  |  | High Gray Level Emphasis | 0.773 [0.517-0.897] |
|  |  | Large Dependence Emphasis | 0.959 [0.909-0.982] |
|  |  | Large Dependence Low Gray Level Emphasis | 0.821 [0.595-0.921] |
|  |  | Small Dependence Emphasis | 0.869 [0.729-0.940] |
|  |  | Small Dependence High Gray Level Emphasis | 0.915 [0.729-0.967] |
| **Wavelet-LHH** | **GLRLM** | Gray Level Non Uniformity | 0.994 [0.984-0.997] |
|  |  | Gray Level Non Uniformity Normalized | 0.951 [0.894-0.978] |
|  |  | Gray Level Variance | 0.943 [0.877-0.975] |
|  |  | High Gray Level Run Emphasis | 0.776 [0.519-0.899] |
|  |  | Long Run Emphasis | 0.963 [0.918-0.984] |
|  |  | Long Run Low Gray Level Emphasis | 0.752 [0.482-0.886] |
|  |  | Run Entropy | 0.908 [0.802-0.958] |
|  |  | Run Length Non Uniformity | 0.984 [0.964-0.993] |
|  |  | Run Length Non Uniformity Normalized | 0.951 [0.893-0.978] |
|  |  | Run Percentage | 0.956 [0.903-0.981] |
|  |  | Run Variance | 0.961 [0.914-0.982] |
|  |  | Short Run Emphasis | 0.954 [0.897-0.979] |
|  |  | Short Run High Gray Level Emphasis | 0.818 [0.585-0.920] |
| **Wavelet-LHH** | **GLSZM** | Gray Level Non Uniformity | 0.907 [0.801-0.958] |
|  |  | Gray Level Non Uniformity Normalized | 0.932 [0.854-0.969] |
|  |  | Gray Level Variance | 0.934 [0.858-0.970] |
|  |  | High Gray Level Zone Emphasis | 0.835 [0.471-0.938] |
|  |  | Large Area Emphasis | 0.963 [0.919-0.984] |
|  |  | Large Area Low Gray Level Emphasis | 0.857 [0.675-0.937] |
|  |  | Size Zone Non Uniformity | 0.858 [0.705-0.935] |
|  |  | Size Zone Non Uniformity Normalized | 0.784 [0.571-0.899] |
|  |  | Small Area High Gray Level Emphasis | 0.867 [0.660-0.944] |
|  |  | Zone Variance | 0.965 [0.923-0.984] |
| **Wavelet-LHH** | **NGTDM** | Busyness | 0.874 [0.670-0.948] |
|  |  | Coarseness | 0.991 [0.978-0.996] |
|  |  | Complexity | 0.938 [0.787-0.977] |
|  |  | Strength | 0.931 [0.796-0.973] |
| **Wavelet-LHL** | **First Order** | 10percentile | 0.939 [0.869-0.973] |
|  |  | 90percentile | 0.935 [0.859-0.971] |
|  |  | Energy | 0.951 [0.892-0.978] |
|  |  | Entropy | 0.942 [0.874-0.974] |
|  |  | Interquartile Range | 0.937 [0.862-0.972] |
|  |  | Maximum | 0.833 [0.658-0.923] |
|  |  | Mean Absolute Deviation | 0.940 [0.871-0.973] |
|  |  | Minimum | 0.852 [0.696-0.932] |
|  |  | Range | 0.919 [0.826-0.963] |
|  |  | Robust Mean Absolute Deviation | 0.936 [0.861-0.971] |
|  |  | Root Mean Squared | 0.940 [0.870-0.973] |
|  |  | Total Energy | 0.951 [0.892-0.978] |
|  |  | Uniformity | 0.946 [0.883-0.976] |
|  |  | Variance | 0.938 [0.867-0.972] |
| **Wavelet-LHL** | **GLCM** | Autocorrelation | 0.869 [0.729-0.940] |
|  |  | Cluster Prominence | 0.923 [0.834-0.965] |
|  |  | Cluster Tendency | 0.930 [0.850-0.969] |
|  |  | Contrast | 0.936 [0.860-0.971] |
|  |  | Correlation | 0.893 [0.771-0.951] |
|  |  | Difference Average | 0.939 [0.869-0.973] |
|  |  | Difference Entropy | 0.942 [0.874-0.974] |
|  |  | Difference Variance | 0.929 [0.847-0.968] |
|  |  | Id | 0.939 [0.867-0.973] |
|  |  | Idm | 0.939 [0.867-0.972] |
|  |  | Imc2 | 0.805 [0.610-0.909] |
|  |  | Inverse Variance | 0.939 [0.868-0.973] |
|  |  | Joint Average | 0.840 [0.673-0.926] |
|  |  | Joint Energy | 0.945 [0.881-0.975] |
|  |  | Joint Entropy | 0.939 [0.868-0.972] |
|  |  | Maximum Probability | 0.924 [0.838-0.966] |
|  |  | Mcc | 0.762 [0.533-0.887] |
|  |  | Sum Average | 0.840 [0.673-0.926] |
|  |  | Sum Entropy | 0.932 [0.854-0.969] |
|  |  | Sum Squares | 0.933 [0.855-0.970] |
| **Wavelet-LHL** | **GLDM** | Dependence Entropy | 0.822 [0.634-0.918] |
|  |  | Dependence Non Uniformity | 0.966 [0.925-0.985] |
|  |  | Dependence Non Uniformity Normalized | 0.890 [0.768-0.950] |
|  |  | Dependence Variance | 0.871 [0.729-0.941] |
|  |  | Gray Level Non Uniformity | 0.983 [0.961-0.992] |
|  |  | Gray Level Variance | 0.938 [0.866-0.972] |
|  |  | High Gray Level Emphasis | 0.874 [0.739-0.942] |
|  |  | Large Dependence Emphasis | 0.929 [0.847-0.968] |
|  |  | Large Dependence Low Gray Level Emphasis | 0.785 [0.571-0.899] |
|  |  | Low Gray Level Emphasis | 0.778 [0.559-0.896] |
|  |  | Small Dependence Emphasis | 0.944 [0.878-0.975] |
|  |  | Small Dependence High Gray Level Emphasis | 0.933 [0.855-0.970] |
| **Wavelet-LHL** | **GLRLM** | Gray Level Non Uniformity | 0.988 [0.972-0.995] |
|  |  | Gray Level Non Uniformity Normalized | 0.948 [0.886-0.976] |
|  |  | Gray Level Variance | 0.939 [0.868-0.973] |
|  |  | High Gray Level Run Emphasis | 0.874 [0.737-0.942] |
|  |  | Long Run Emphasis | 0.943 [0.875-0.974] |
|  |  | Long Run High Gray Level Emphasis | 0.809 [0.616-0.911] |
|  |  | Long Run Low Gray Level Emphasis | 0.781 [0.563-0.897] |
|  |  | Low Gray Level Run Emphasis | 0.777 [0.556-0.895] |
|  |  | Run Entropy | 0.925 [0.835-0.966] |
|  |  | Run Length Non Uniformity | 0.983 [0.962-0.992] |
|  |  | Run Length Non Uniformity Normalized | 0.937 [0.862-0.972] |
|  |  | Run Percentage | 0.935 [0.858-0.971] |
|  |  | Run Variance | 0.938 [0.864-0.972] |
|  |  | Short Run Emphasis | 0.941 [0.870-0.973] |
|  |  | Short Run High Gray Level Emphasis | 0.886 [0.760-0.948] |
|  |  | Short Run Low Gray Level Emphasis | 0.774 [0.551-0.894] |
| **Wavelet-LHL** | **GLSZM** | Gray Level Non Uniformity | 0.954 [0.898-0.979] |
|  |  | Gray Level Non Uniformity Normalized | 0.901 [0.791-0.955] |
|  |  | Gray Level Variance | 0.934 [0.858-0.970] |
|  |  | High Gray Level Zone Emphasis | 0.884 [0.756-0.947] |
|  |  | Large Area Emphasis | 0.949 [0.888-0.977] |
|  |  | Size Zone Non Uniformity | 0.943 [0.876-0.975] |
|  |  | Small Area High Gray Level Emphasis | 0.887 [0.761-0.949] |
|  |  | Zone Entropy | 0.752 [0.516-0.882] |
|  |  | Zone Percentage | 0.932 [0.853-0.970] |
|  |  | Zone Variance | 0.950 [0.890-0.978] |
| **Wavelet-LHL** | **NGTDM** | Coarseness | 0.989 [0.975-0.995] |
|  |  | Complexity | 0.934 [0.857-0.970] |
|  |  | Strength | 0.911 [0.811-0.960] |
| **Wavelet-Llh** | **First Order** | 10percentile | 0.960 [0.913-0.982] |
|  |  | 90percentile | 0.966 [0.926-0.985] |
|  |  | Energy | 0.962 [0.917-0.983] |
|  |  | Entropy | 0.965 [0.923-0.984] |
|  |  | Interquartile Range | 0.964 [0.922-0.984] |
|  |  | Mean Absolute Deviation | 0.966 [0.926-0.985] |
|  |  | Robust Mean Absolute Deviation | 0.962 [0.917-0.983] |
|  |  | Root Mean Squared | 0.966 [0.924-0.985] |
|  |  | Total Energy | 0.962 [0.917-0.983] |
|  |  | Uniformity | 0.964 [0.921-0.984] |
|  |  | Variance | 0.968 [0.929-0.986] |
| **Wavelet-Llh** | **GLCM** | Autocorrelation | 0.809 [0.617-0.911] |
|  |  | Cluster Prominence | 0.968 [0.928-0.986] |
|  |  | Cluster Tendency | 0.973 [0.941-0.988] |
|  |  | Contrast | 0.965 [0.910-0.985] |
|  |  | Correlation | 0.877 [0.727-0.945] |
|  |  | Difference Average | 0.968 [0.924-0.986] |
|  |  | Difference Entropy | 0.969 [0.926-0.986] |
|  |  | Difference Variance | 0.958 [0.894-0.982] |
|  |  | Id | 0.966 [0.922-0.985] |
|  |  | Idm | 0.967 [0.925-0.985] |
|  |  | Imc1 | 0.847 [0.670-0.931] |
|  |  | Imc2 | 0.865 [0.673-0.942] |
|  |  | Inverse Variance | 0.971 [0.912-0.989] |
|  |  | Joint Average | 0.783 [0.571-0.897] |
|  |  | Joint Energy | 0.967 [0.926-0.985] |
|  |  | Joint Entropy | 0.970 [0.934-0.987] |
|  |  | Maximum Probability | 0.957 [0.907-0.981] |
|  |  | Mcc | 0.790 [0.553-0.905] |
|  |  | Sum Average | 0.783 [0.571-0.897] |
|  |  | Sum Entropy | 0.971 [0.935-0.987] |
|  |  | Sum Squares | 0.972 [0.935-0.988] |
| **Wavelet-Llh** | **GLDM** | Dependence Entropy | 0.925 [0.839-0.966] |
|  |  | Dependence Non Uniformity | 0.980 [0.955-0.991] |
|  |  | Dependence Non Uniformity Normalized | 0.947 [0.885-0.976] |
|  |  | Dependence Variance | 0.937 [0.864-0.972] |
|  |  | Gray Level Non Uniformity | 0.988 [0.971-0.995] |
|  |  | Gray Level Variance | 0.969 [0.932-0.986] |
|  |  | High Gray Level Emphasis | 0.827 [0.650-0.920] |
|  |  | Large Dependence Emphasis | 0.959 [0.911-0.982] |
|  |  | Large Dependence Low Gray Level Emphasis | 0.818 [0.632-0.915] |
|  |  | Small Dependence Emphasis | 0.922 [0.833-0.965] |
|  |  | Small Dependence High Gray Level Emphasis | 0.942 [0.872-0.974] |
| **Wavelet-Llh** | **GLRLM** | Gray Level Non Uniformity | 0.990 [0.976-0.996] |
|  |  | Gray Level Non Uniformity Normalized | 0.963 [0.919-0.983] |
|  |  | Gray Level Variance | 0.967 [0.928-0.985] |
|  |  | High Gray Level Run Emphasis | 0.830 [0.656-0.921] |
|  |  | Long Run Emphasis | 0.960 [0.912-0.982] |
|  |  | Run Entropy | 0.954 [0.899-0.979] |
|  |  | Run Length Non Uniformity | 0.985 [0.966-0.993] |
|  |  | Run Length Non Uniformity Normalized | 0.956 [0.904-0.980] |
|  |  | Run Percentage | 0.961 [0.913-0.982] |
|  |  | Run Variance | 0.958 [0.909-0.981] |
|  |  | Short Run Emphasis | 0.956 [0.904-0.980] |
|  |  | Short Run High Gray Level Emphasis | 0.865 [0.721-0.938] |
| **Wavelet-Llh** | **GLSZM** | Gray Level Non Uniformity | 0.958 [0.909-0.981] |
|  |  | Gray Level Non Uniformity Normalized | 0.897 [0.784-0.953] |
|  |  | Gray Level Variance | 0.948 [0.886-0.977] |
|  |  | High Gray Level Zone Emphasis | 0.890 [0.769-0.950] |
|  |  | Large Area Emphasis | 0.951 [0.893-0.978] |
|  |  | Large Area High Gray Level Emphasis | 0.821 [0.634-0.917] |
|  |  | Large Area Low Gray Level Emphasis | 0.905 [0.799-0.957] |
|  |  | Size Zone Non Uniformity | 0.880 [0.747-0.945] |
|  |  | Small Area High Gray Level Emphasis | 0.907 [0.802-0.958] |
|  |  | Zone Percentage | 0.871 [0.732-0.941] |
|  |  | Zone Variance | 0.953 [0.897-0.979] |
| **Wavelet-Llh** | **NGTDM** | Busyness | 0.814 [0.624-0.913] |
|  |  | Coarseness | 0.989 [0.976-0.995] |
|  |  | Complexity | 0.806 [0.593-0.911] |
|  |  | Strength | 0.842 [0.616-0.933] |
| **Wavelet-LLL** | **First Order** | 10percentile | 1.000 [1.000-1.000] |
|  |  | 90percentile | 1.000 [1.000-1.000] |
|  |  | Entropy | 0.951 [0.894-0.978] |
|  |  | Interquartile Range | 0.969 [0.932-0.986] |
|  |  | Kurtosis | 0.967 [0.927-0.985] |
|  |  | Maximum | 1.000 [1.000-1.000] |
|  |  | Mean | 1.000 [1.000-1.000] |
|  |  | Mean Absolute Deviation | 0.962 [0.917-0.983] |
|  |  | Median | 1.000 [1.000-1.000] |
|  |  | Minimum | 0.999 [0.998-1.000] |
|  |  | Range | 0.936 [0.862-0.971] |
|  |  | Robust Mean Absolute Deviation | 0.970 [0.932-0.986] |
|  |  | Root Mean Squared | 1.000 [1.000-1.000] |
|  |  | Skewness | 0.819 [0.633-0.916] |
|  |  | Uniformity | 0.962 [0.917-0.983] |
|  |  | Variance | 0.965 [0.924-0.984] |
| **Wavelet-LLL** | **GLCM** | Autocorrelation | 0.933 [0.855-0.970] |
|  |  | Cluster Prominence | 0.976 [0.948-0.989] |
|  |  | Cluster Shade | 0.909 [0.807-0.959] |
|  |  | Cluster Tendency | 0.961 [0.914-0.982] |
|  |  | Contrast | 0.948 [0.887-0.977] |
|  |  | Correlation | 0.769 [0.545-0.891] |
|  |  | Difference Average | 0.948 [0.886-0.977] |
|  |  | Difference Entropy | 0.947 [0.884-0.976] |
|  |  | Difference Variance | 0.945 [0.879-0.975] |
|  |  | Id | 0.952 [0.894-0.978] |
|  |  | Idm | 0.952 [0.895-0.979] |
|  |  | Idmn | 0.754 [0.517-0.884] |
|  |  | Idn | 0.812 [0.621-0.913] |
|  |  | Imc1 | 0.833 [0.657-0.923] |
|  |  | Imc2 | 0.830 [0.652-0.922] |
|  |  | Inverse Variance | 0.943 [0.874-0.974] |
|  |  | Joint Average | 0.876 [0.738-0.943] |
|  |  | Joint Energy | 0.966 [0.926-0.985] |
|  |  | Joint Entropy | 0.948 [0.886-0.976] |
|  |  | Maximum Probability | 0.973 [0.940-0.988] |
|  |  | Mcc | 0.803 [0.606-0.908] |
|  |  | Sum Average | 0.876 [0.738-0.943] |
|  |  | Sum Entropy | 0.940 [0.870-0.973] |
|  |  | Sum Squares | 0.961 [0.914-0.982] |
| **Wavelet-LLL** | **GLDM** | Dependence Entropy | 0.876 [0.726-0.945] |
|  |  | Dependence Non Uniformity | 0.974 [0.942-0.988] |
|  |  | Dependence Non Uniformity Normalized | 0.943 [0.876-0.975] |
|  |  | Dependence Variance | 0.951 [0.892-0.978] |
|  |  | Gray Level Non Uniformity | 0.984 [0.964-0.993] |
|  |  | Gray Level Variance | 0.964 [0.922-0.984] |
|  |  | High Gray Level Emphasis | 0.935 [0.859-0.971] |
|  |  | Large Dependence Emphasis | 0.963 [0.917-0.983] |
|  |  | Large Dependence High Gray Level Emphasis | 0.911 [0.811-0.960] |
|  |  | Small Dependence Emphasis | 0.953 [0.896-0.979] |
|  |  | Small Dependence High Gray Level Emphasis | 0.963 [0.917-0.983] |
| **Wavelet-LLL** | **GLRLM** | Gray Level Non Uniformity | 0.987 [0.970-0.994] |
|  |  | Gray Level Non Uniformity Normalized | 0.958 [0.909-0.981] |
|  |  | Gray Level Variance | 0.963 [0.920-0.984] |
|  |  | High Gray Level Run Emphasis | 0.936 [0.860-0.971] |
|  |  | Long Run Emphasis | 0.965 [0.923-0.985] |
|  |  | Long Run High Gray Level Emphasis | 0.924 [0.836-0.966] |
|  |  | Run Entropy | 0.935 [0.857-0.971] |
|  |  | Run Length Non Uniformity | 0.984 [0.964-0.993] |
|  |  | Run Length Non Uniformity Normalized | 0.952 [0.894-0.979] |
|  |  | Run Percentage | 0.957 [0.904-0.981] |
|  |  | Run Variance | 0.969 [0.931-0.986] |
|  |  | Short Run Emphasis | 0.954 [0.899-0.979] |
|  |  | Short Run High Gray Level Emphasis | 0.939 [0.866-0.973] |
| **Wavelet-LLL** | **GLSZM** | Gray Level Non Uniformity | 0.940 [0.870-0.973] |
|  |  | Gray Level Non Uniformity Normalized | 0.921 [0.830-0.964] |
|  |  | Gray Level Variance | 0.938 [0.864-0.972] |
|  |  | High Gray Level Zone Emphasis | 0.945 [0.880-0.976] |
|  |  | Large Area Emphasis | 0.975 [0.945-0.989] |
|  |  | Large Area High Gray Level Emphasis | 0.956 [0.903-0.980] |
|  |  | Large Area Low Gray Level Emphasis | 0.822 [0.640-0.917] |
|  |  | Size Zone Non Uniformity | 0.910 [0.808-0.959] |
|  |  | Small Area High Gray Level Emphasis | 0.934 [0.856-0.970] |
|  |  | Zone Entropy | 0.803 [0.607-0.908] |
|  |  | Zone Percentage | 0.959 [0.910-0.982] |
|  |  | Zone Variance | 0.975 [0.945-0.989] |
| **Wavelet-LLL** | **NGTDM** | Coarseness | 0.990 [0.979-0.996] |
|  |  | Complexity | 0.956 [0.904-0.980] |
|  |  | Contrast | 0.895 [0.778-0.952] |
|  |  | Strength | - 1. .936-0.987] |

**S1.3 Radiomics feature selection**

To Remove the unbalance of the training data set, we used the Synthetic Minority Oversampling TEchnique (SMOTE) to make positive/negative samples balance. We applied the normalization on the feature matrix. For each feature vector, we calculated the mean value and the standard deviation. Each feature vector was subtracted by the mean value and was divided by the standard deviation. After normalization process, each vector has zero center and unit standard deviation. Since the dimension of feature space was high, we compared the similarity of each feature pair. If the PCC value of the feature pair was larger than 0.990, we removed one of them. After this process, the dimension of the feature space was reduced and each feature was independent to each other. Before building the model, we used recursive feature elimination (RFE) to select features. The goal of RFE is to select features based on a classifier by recursively considering smaller set of the features.

**S1.4 Model building and testing**

To determine the hyper-parameter (e.g. the number of features, classifier, etc.) of models, we applied cross validation with 5-fold on the training set. The hyper-parameters were set according to the model performance on the validation set. The performance of the model was evaluated using receiver operating characteristic (ROC) curve analysis. The area under the ROC curve (AUC) was calculated for quantification. The accuracy, sensitivity and specificity were also calculated at a cutoff value that maximized the value of the Yorden index. The model with the highest Youden index in internal cross validation was selected as the optimal model, because the aim of this study was to distinguish patients with different PNAHS risk. We also estimated the 95% confidence interval by bootstrape with 1000 samples. All above processes were implemented with FeAture Explorer Pro (FAE, V 0.5.12) on Python (3.7.6).

**S1.5 Selected features in the radiomics model**

In the optimal radiomics model with the highest Youden index in the cross validation, logistic regression was used as the classifier, which is a linear classifier that combines all the features. Four features were selected:

1. **IMG_original_shape_Maximum2DDiameterSlice**: This is a morphological descriptor. It quantifies the longest diameter of the contiguous tissue region within the standardized VOI. This feature reflects the maximum axial diameter of the contiguous region formed by voxels within the standardized VOI that correspond to the conventional gray-scale values of liver tissue. Subtle abnormal imaging findings, such as fat deposition, dilated microvessels or tiny nodules—even those not easily detectable or quantifiable by the human eye—can influence the value of this feature.
2. **IMG_wavelet-HHH_glszm_GrayLevelNonUniformity**: This feature was calculated in the high-frequency ("HHH") wavelet domain, this feature measures the non-uniformity of gray-level intensity distribution. A higher value indicates greater disparity in pixel intensities across the VOI. Therefore, this is a key metric of textural heterogeneity. In liver imaging, significantly elevated values may suggest a patchy or inhomogeneous tissue composition. This could directly correspond to non-uniform fat deposition, scattered inflammatory foci, or mixed-density areas resulting from early fibrotic changes, all of which disrupt the homogeneity of normal liver parenchyma.
3. **IMG_wavelet-HHH_glszm_SizeZoneNonUniformityNormalized**: Also derived from the "HHH" (high frequency in three dimensions) domain, this feature measures the non-uniformity in the size distribution of contiguous homogeneous regions (zones). A higher value indicates that zones of similar intensity vary greatly in size. Early, diffuse, and uniform microstructural changes—characterized by increased homogeneity in high-frequency texture—may serve as an important precursor signal for the insidious onset of fat infiltration. This complements other features, such as the positive coefficient of GrayLevelNonUniformity: while the latter likely reflects more pronounced disparities in gray-level intensity, the former sensitively captures a subtle homogenization trend in structural distribution.
4. **IMG_wavelet-LHH_glcm_Imc1**: "Informational Measure of Correlation 1" is calculated in the "LHH" domain (a combination of high and low frequency in different dimensions). It assesses the complexity and linear dependency between pixel pairs, reflecting the overall orderliness or predictability of the texture pattern. This feature may capture underlying tissue microstructure patterns. A shift towards a more regular microstructure, such as the organized, directional deposition of collagen fibers due to inflammation, could increase the linear dependency and order in the image texture, thereby altering the Imc1 value. It is therefore considered a potential indicator for connective tissue remodeling.

The coefficients of each selected feature are demonstrated in **Table s2**.

**Table S2. Coefficients of the Features in the Radiomics Model**

| Features | Coefficients |  |
| --- | --- | --- |
| IMG_original_shape_Maximum2DDiameterSlice | 0.316 |  |
| IMG_wavelet-HHH_glszm_GrayLevelNonUniformity | 0.980 |  |
| IMG_wavelet-HHH_glszm_SizeZoneNonUniformityNormalized | -0.453 |  |
| IMG_wavelet-LHH_glcm_Imc1 | 0.264 |  |

**Table S3. Baseline Characteristics of the Patients in the Training Set and the Testing Set**

|  | Training set  (n = 219) | Testing set  (n = 88) | P-value | |
| --- | --- | --- | --- | --- |
| Sex (male，n) | 153 (69.9 %) | 66 (75.0 %) | 0.405 | |
| Age (year) | 51.00 (42.00-59.00) | 54.00 (44.00-63.00) | 0.139 | |
| BMI (kg/m^2^) | 18.67 (16.65-20.70) | 18.86 (17.01-20.03) | 0.845 | |
| REE (kcal/d) | 1300.00 (1125.00-1473.00) | 1313.00 (1175.00-1453.00) | 0.633 | |
| NRS2002 ≥ 5 (n) | 95 (32.8 %) | 46 (52.3 %) | 0.166 | |
| GLIM - severe malnutrition (n) | 99 (45.2 %) | 40 (45.4 %) | 1.000 | |
| SBS types* |  |  | 0.475 | |
| I (n) | 60 (28.7 %) | 24 (30.0 %) |  | |
| II (n) | 74 (35.4 %) | 32 (40.0 %) |  | |
| III (n) | 75 (35.9 %) | 24 (30.0 %) |  | |
| Underlying diseases |  |  |  | |
| Hypertension (n) | 56 (25.6 %) | 23 (26.1%) | 1.000 |  |
| Diabetes (n) | 29 (13.2 %) | 12 (13.6 %) | 1.000 |  |
| Thrombosis (n) | 81 (37.0 %) | 34 (38.6 %) | 0.796 |  |
| ALT (U/L) | 28.00 (17.00-39.00) | 27.00 (12.00-39.00) | 0.228 | |
| AST (U/L) | 24.00 (16.00-32.00) | 22.00 (15.00-30.00) | 0.412 | |
| ALP (U/L) | 90.00 (63.00-119.00) | 86.00 (56.00-120.00) | 0.419 | |
| GGT (U/L) | 29.00 (16.00-43.00) | 31.00 (19.00-49.00) | 0.138 | |
| TBIL (μmol/L) | 12.40 (8.40-17.10) | 13.70 (8.90-18.00) | 0.179 | |
| DBIL (μmol/L) | 5.30 (3.40-7.20) | 5.20 (3.10-7.20) | 0.772 | |
| IBIL (μmol/L) | 6.70 (3.90-10.40) | 7.30 (4.60-12.10) | 0.181 | |
| Creatinine (μmol/L) | 64.30 (42.50-86.60) | 61.40 (43.10-78.90) | 0.559 | |
| Urea (mmol/L) | 7.00 (4.60-11.10) | 6.45 (3.30-10.40) | 0.194 | |
| TG (mmol/L) | 1.53 (0.92-2.02) | 1.23 (0.80-1.93) | 0.092 | |
| TC (mmol/L) | 2.89 (2.34-3.64) | 2.89 (2.08-3.46) | 0.271 | |
| Ratio of glucose to lipid of PN | 1.20 (1.00-1.51) | 1.00 (0.85-1.40) | 0.114 | |
| Ratio of fish oil to lipid of PN | 0.17 (0.00-0.25) | 0.16 (0.00-0.25) | 0.628 | |
| Frequency of PN (d/w) | 7.00 (3.00-7.00) | 5.00 (3.00-7.00) | 0.487 | |
| L3-muscle area (mm^2^) | 91.92 (76.96-106.61) | 95.63 (73.05-106.94) | 0.682 | |
| L3-intermuscular fat area (mm^2^) | 609.25 (356.14-1109.61) | 672.51 (413.73-1170.76) | 0.698 | |
| Sarcopenia (n) | 180 (82.2%) | 68 (77.2%) | 0.323 | |

Note: Continuous variables are reported as medians and IQRs, while categorical variables are reported as numbers and percentages. BMI = body mass index, REE = resting energy expense, NRS2002 = nutrition risk screening 2002, GLIM = Global Leadership Initiative on Malnutrition, SBS = short bowel syndrome, ALT = alanine aminotransferase, AST = aspartate aminotransferase, ALP = alkaline phosphatase, GGT = γ(gamma) - glutamyl transpeptidase, TBIL = total bilirubin, DBIL = direct bilirubin, IBIL= indirect bilirubin, TG = triglycerides, TC = total cholesterol.

*The chronic intestinal failure of 18 patients is not caused by short bowel syndrome.

**Table S4.** **Baseline Characteristics of the PNAHS-positive and PNAHS-negative Patients in the Training and Testing Sets**

|  | Training set (n = 219) | | | Testing set (n = 88) | | |
| --- | --- | --- | --- | --- | --- | --- |
|  | PNAHS +  (n = 57) | PNAHS –  (n = 162) | P-value | PNAHS +  (n = 26) | PNAHS –  (n = 62) | P-value |
| Sex (male，n) | 48 (84.2 %) | 105 (64.8 %) | 0.311 | 17 (65.4 %) | 49 (79.0 %) | 0.280 |
| Age (year) | 53.00 (39.00-61.00) | 50.00 (43.00-58.00) | 0.310 | 60.00 (53.00-69.00) | 51.00 (39.00-60.00) | 0.005 |
| BMI (kg/m^2^) | 18.89 (16.19-21.08) | 18.67 (16.80-20.51) | 0.865 | 18.78 (16.90-20.06) | 18.89 (17.30-20.03) | 0.735 |
| REE (kcal/d) | 1300.00 (1125.00-1475.00) | 1300.00 (1125.00-1460.00) | 0.965 | 1280.00 (1187.50-1375.00) | 1315.00 (1175.00-1487.50) | 0.303 |
| NRS-2002 ≥ 5 (n) | 23 (40.4 %) | 72 (44.4 %) | 0.643 | 13 (50.0 %) | 33 (53.2 %) | 0.818 |
| GLIM-severe malnutrition (n) | 34 (59.6 %) | 86 (53.1 %) | 0.441 | 14 (53.8 %) | 34 (54.8 %) | 1.000 |
| SBS types* |  |  | 0.796 |  |  | 0.643 |
| I (n) | 16 (29.6 %) | 44 (28.4 %) |  | 5 (20.0 %) | 19 (34.5 %) |  |
| II (n) | 17 (31.5 %) | 57 (36.8 %) |  | 13 (52.0 %) | 19 (34.5 %) |  |
| III (n) | 21 (38.9 %) | 54 (34.8 %) |  | 7 (28.0 %) | 17 (31.0 %) |  |
| Underlying diseases |  |  |  |  |  |  |
| Hypertension (n) | 19 (33.3 %) | 37 (22.8 %) | 0.157 | 9 (34.6 %) | 14 (22.6 %) | 0.291 |
| Diabetes (n) | 8 (14.0 %) | 21 (13.0 %) | 1.000 | 3 (11.5 %) | 9 (14.5 %) | 0.754 |
| Thrombosis (n) | 22 (38.6 %) | 59 (36.4 %) | 0.873 | 11 (42.3 %) | 23 (37.1 %) | 0.811 |
| ALT (U/L) | 27.00 (16.00-41.00) | 28.00 (18.00-39.00) | 0.663 | 28.00 (10.00-40.00) | 27.00 (14.00-39.00) | 0.516 |
| AST (U/L) | 24.00 (16.00-30.00) | 23.00 (16.00-32.00) | 0.950 | 24.00 (11.00-39.00) | 21.00 (15.00-29.00) | 0.310 |
| ALP (U/L) | 88.00 (65.00-134.00) | 90.00 (61.00-117.00) | 0.300 | 73.00 (52.00-132.00) | 86.00 (61.00-118.00) | 0.905 |
| GGT (U/L) | 32.00 (19.00-45.00) | 27.00 (15.00-42.00) | 0.194 | 31.00 (22.00-49.00) | 31.00 (19.00-49.00) | 0.884 |
| TBIL (μmol/L) | 13.50 (10.60-18.20) | 11.70 (8.00-16.40) | 0.051 | 15.90 (8.80-23.30) | 12.90 (9.20-17.10) | 0.091 |
| DBIL (μmol/L) | 5.60 (4.20-6.60) | 5.00 (3.10-7.40) | 0.439 | 5.70 (3.20-8.70) | 4.70 (3.10-6.50) | 0.224 |
| IBIL (μmol/L) | 8.60 (5.00-10.50) | 6.10 (3.70-10.30) | 0.070 | 8.50 (2.80-16.20) | 7.10 (5.00-11.10) | 0.418 |
| Creatinine (μmol/L) | 81.00 (57.00-105.20) | 59.00 (41.80-82.90) | < 0.001 | 73.40 (50.00-89.70) | 56.70 (3.30-9.30) | 0.036 |
| Urea (mmol/L) | 13.20 (7.60-31.10) | 6.50 (4.30-8.60) | < 0.001 | 8.90 (5.80-23.40) | 4.90 (53.00-69.00) | 0.004 |
| TG (mmol/L) | 1.63 (1.16-2.39) | 1.42 (0.84-1.95) | 0.034 | 1.10 (0.60-1.87) | 1.25 (0.81-1.99) | 0.478 |
| TC (mmol/L) | 3.54 (2.48-4.48) | 2.83 (2.19-3.40) | <0.001 | 2.61 (1.97-3.70) | 2.89 (2.09-3.44) | 0.437 |
| Ratio of glucose to lipid of PN | 1.27 (1.00-1.65) | 1.17 (1.00-1.40) | 0.279 | 1.14 (0.78-1.46) | 1.00 (0.94-1.40) | 0.861 |
| Ratio of fish oil to lipid of PN | 0.15 (0.00-0.25) | 0.17 (0.00-0.25) | 0.463 | 0.15 (0.00-0.24) | 0.16 (0.00-0.25) | 0.860 |
| Frequency of PN (d/w) | 7.00 (7.00-7.00) | 5.00 (3.00-7.00) | < 0.001 | 7.00 (3.00-7.00) | 4.00 (3.00-7.00) | 0.003 |
| L3-muscle area (mm^2^) | 88.77 (72.76-104.33) | 93.79 (78.42-108.40) | 0.191 | 95.42 (67.96-107.55) | 91.43 (73.77-105.11) | 0.841 |
| L3-intermuscular fat area (mm^2^) | 1222.17 (682.27-2626.34) | 525.72 (316.06-869.02) | < 0.001 | 873.87 (646.30-1560.37) | 550.08 (370.78-971.47) | 0.016 |
| Sarcopenia (n) | 46 (80.7 %) | 134 (82.7 %) | 0.841 | 19 (73.1%) | 49 (79.0 %) | 0.545 |

Note: Continuous variables are reported as medians and IQRs, while categorical variables are reported as numbers and percentages. BMI = body mass index, REE = resting energy expense, NRS2002 = nutrition risk screening 2002, GLIM = Global Leadership Initiative on Malnutrition, SBS = short bowel syndrome, ALT = alanine aminotransferase, AST = aspartate aminotransferase, ALP = alkaline phosphatase, GGT = γ(gamma) - glutamyl transpeptidase, TBIL = total bilirubin, DBIL = direct bilirubin, IBIL= indirect bilirubin, TG = triglycerides, TC = total cholesterol.

*The chronic intestinal failure of 18 patients is not caused by short bowel syndrome.

**Table S5.** **Performance of Models in 5-Fold Cross Validation**

| Model | Fold | Sensitivity | Specificity | Accuracy | Youden index | AUC | P-value^a^ |
| --- | --- | --- | --- | --- | --- | --- | --- |
| Combined model | 1 | 0.929 | 0.759 | 0.814 | 0.687 | 0.872 |  |
|  | 2 | 0.750 | 1.000 | 0.932 | 0.750 | 0.859 |  |
|  | 3 | 1.000 | 0.789 | 0.818 | 0.789 | 0.943 |  |
|  | 4 | 0.813 | 0.964 | 0.909 | 0.777 | 0.942 |  |
|  | 5 | 0.889 | 0.771 | 0.795 | 0.660 | 0.873 |  |
|  | **Mean** | 0.876 | 0.857 | 0.854 | 0.733 | 0.898 | NA |
| Radiomics model | 1 | 0.429 | 0.966 | 0.791 | 0.394 | 0.719 |  |
|  | 2 | 0.667 | 1.000 | 0.909 | 0.667 | 0.815 |  |
|  | 3 | 0.833 | 0.921 | 0.909 | 0.754 | 0.829 |  |
|  | 4 | 1.000 | 0.607 | 0.750 | 0.607 | 0.850 |  |
|  | 5 | 0.778 | 0.800 | 0.795 | 0.578 | 0.746 |  |
|  | **Mean** | 0.741 | 0.859 | 0.831 | 0.600 | 0.792 | 0.011 |
| Clinical model | 1 | 0.786 | 0.793 | 0.791 | 0.579 | 0.847 |  |
|  | 2 | 1.000 | 0.469 | 0.614 | 0.469 | 0.777 |  |
|  | 3 | 0.833 | 0.921 | 0.909 | 0.754 | 0.860 |  |
|  | 4 | 0.813 | 0.786 | 0.795 | 0.598 | 0.885 |  |
|  | 5 | 0.889 | 0.600 | 0.659 | 0.489 | 0.743 |  |
|  | **Mean** | 0.864 | 0.714 | 0.754 | 0.578 | 0.822 | 0.075 |
| Unet | 1 | 0.857 | 0.862 | 0.860 | 0.719 | 0.938 |  |
|  | 2 | 1.000 | 0.688 | 0.773 | 0.688 | 0.919 |  |
|  | 3 | 1.000 | 0.895 | 0.909 | 0.895 | 0.947 |  |
|  | 4 | 0.750 | 0.964 | 0.886 | 0.714 | 0.911 |  |
|  | 5 | 1.000 | 0.800 | 0.841 | 0.800 | 0.914 |  |
|  | **Mean** | 0.921 | 0.842 | 0.854 | 0.763 | 0.926 | 0.587 |
| Resnet+XGboostFC | 1 | 0.929 | 0.793 | 0.837 | 0.722 | 0.919 |  |
|  | 2 | 0.833 | 0.781 | 0.795 | 0.615 | 0.884 |  |
|  | 3 | 0.833 | 0.947 | 0.932 | 0.781 | 0.930 |  |
|  | 4 | 0.938 | 0.893 | 0.909 | 0.830 | 0.954 |  |
|  | 5 | 0.889 | 0.743 | 0.773 | 0.632 | 0.873 |  |
|  | **Mean** | 0.921 | 0.842 | 0.854 | 0.763 | 0.926 | <0.001 |
| VIT | 1 | 0.857 | 0.759 | 0.791 | 0.616 | 0.853 |  |
|  | 2 | 0.667 | 0.781 | 0.750 | 0.448 | 0.772 |  |
|  | 3 | 0.833 | 0.921 | 0.909 | 0.754 | 0.895 |  |
|  | 4 | 0.750 | 0.857 | 0.818 | 0.607 | 0.865 |  |
|  | 5 | 0.778 | 0.771 | 0.773 | 0.549 | 0.781 |  |
|  | **Mean** | 0.777 | 0.818 | 0.808 | 0.595 | 0.833 | 0.073 |
| SwinUNTR | 1 | 0.857 | 0.793 | 0.814 | 0.650 | 0.852 |  |
|  | 2 | 0.917 | 0.625 | 0.705 | 0.542 | 0.797 |  |
|  | 3 | 0.833 | 0.921 | 0.909 | 0.754 | 0.860 |  |
|  | 4 | 0.875 | 0.750 | 0.795 | 0.625 | 0.888 |  |
|  | 5 | 0.889 | 0.571 | 0.636 | 0.460 | 0.746 |  |
|  | **Mean** | 0.874 | 0.732 | 0.772 | 0.606 | 0.829 | 0.001 |
| Densenet121 | 1 | 0.643 | 1.000 | 0.884 | 0.643 | 0.865 |  |
|  | 2 | 0.833 | 0.656 | 0.705 | 0.490 | 0.781 |  |
|  | 3 | 1.000 | 0.658 | 0.705 | 0.658 | 0.899 |  |
|  | 4 | 0.875 | 0.964 | 0.932 | 0.839 | 0.940 |  |
|  | 5 | 0.889 | 0.686 | 0.727 | 0.575 | 0.775 |  |
|  | **Mean** | 0.848 | 0.793 | 0.790 | 0.641 | 0.852 | 0.543 |
| Resnet18 | 1 | 0.714 | 0.931 | 0.860 | 0.645 | 0.857 |  |
|  | 2 | 0.750 | 0.719 | 0.727 | 0.469 | 0.784 |  |
|  | 3 | 0.833 | 0.789 | 0.795 | 0.623 | 0.882 |  |
|  | 4 | 0.875 | 0.786 | 0.818 | 0.661 | 0.891 |  |
|  | 5 | 0.667 | 0.800 | 0.773 | 0.467 | 0.743 |  |
|  | **Mean** | 0.768 | 0.805 | 0.795 | 0.573 | 0.831 | 0.158 |
| CNN | 1 | 0.643 | 0.931 | 0.837 | 0.574 | 0.835 |  |
|  | 2 | 0.917 | 0.688 | 0.750 | 0.604 | 0.828 |  |
|  | 3 | 0.833 | 0.921 | 0.909 | 0.754 | 0.860 |  |
|  | 4 | 0.750 | 0.857 | 0.818 | 0.607 | 0.868 |  |
|  | 5 | 0.889 | 0.600 | 0.659 | 0.489 | 0.730 |  |
|  | **Mean** | 0.806 | 0.799 | 0.795 | 0.606 | 0.824 | 0.067 |
| MLP | 1 | 0.857 | 0.931 | 0.907 | 0.788 | 0.929 |  |
|  | 2 | 0.917 | 0.563 | 0.659 | 0.479 | 0.737 |  |
|  | 3 | 0.833 | 0.921 | 0.909 | 0.754 | 0.882 |  |
|  | 4 | 0.813 | 0.821 | 0.818 | 0.634 | 0.897 |  |
|  | 5 | 0.889 | 0.800 | 0.818 | 0.689 | 0.832 |  |
|  | **Mean** | 0.862 | 0.807 | 0.822 | 0.669 | 0.855 | 0.224 |

^a^ The mean AUC of the combined model was compared with that of the other models using t-test. Differences were considered statistically significant at P < 0.05.

**Table S6.** **Calibration Parameters of Models in the Training Set**

| Model | Intercept | Slope | Brier Score | Eavg | P-value^a^ |
| --- | --- | --- | --- | --- | --- |
| Combined model | 0 | 1 | 0.103 | 0.207 | 0.624 |
| Radiomics model | -1.068 | 1.003 | 0.183 | 0.370 | <0.001 |
| Clinical model | 0 | 1 | 0.139 | 0.278 | 0.629 |
| Unet | 0 | 1 | 0.095 | 0.187 | 0.785 |
| ResNet+XGboost/FC | 0 | 1 | 0.097 | 0.195 | 0.951 |
| VIT | 0 | 1 | 0.139 | 0.277 | 0.612 |
| SwinUNETR | 0 | 1 | 0.137 | 0.274 | 0.963 |
| DenseNet121 | 0 | 1 | 0.126 | 0.253 | 0.723 |
| ResNet18 | 0 | 1 | 0.132 | 0.266 | 0.809 |
| CNN | 0 | 1 | 0.138 | 0.277 | 0.889 |
| MLP | 0 | 1 | 0.122 | 0.243 | 0.594 |

^a^ P-value was calculated using Hosmer-Lemeshow test. If the p-value is greater than 0.05, it indicates that the model passes the Hosmer-Lemeshow test, meaning there is no significant discrepancy between the predicted and observed values.

**Table S7. Calibration Parameters of Models in the Testing Set**

| Model | Intercept | Slope | Brier Score | Eavg | P-value^a^ |
| --- | --- | --- | --- | --- | --- |
| Combined model | -0.126 | 0.740 | 0.139 | 0.244 | 0.214 |
| Radiomics model | -1.258 | 1.386 | 0.180 | 0.372 | 0.002 |
| Clinical model | -0.098 | 0.615 | 0.188 | 0.324 | <0.001 |
| Unet | -0.433 | 0.206 | 0.239 | 0.337 | <0.001 |
| ResNet+XGboost/FC | -0.684 | 0.052 | 0.238 | 0.332 | <0.001 |
| VIT | -0.100 | 0.621 | 0.188 | 0.324 | 0.009 |
| SwinUNETR | -0.296 | 0.597 | 0.189 | 0.332 | 0.025 |
| DenseNet121 | -0.254 | 0.498 | 0.200 | 0.333 | 0.002 |
| ResNet18 | -0.117 | 0.620 | 0.187 | 0.321 | 0.2 |
| CNN | -0.052 | 0.604 | 0.190 | 0.321 | 0.004 |
| MLP | -0.306 | 0.420 | 0.202 | 0.322 | <0.001 |

^a^ P-value was calculated using Hosmer-Lemeshow test. If the p-value is greater than 0.05, it indicates that the model passes the Hosmer-Lemeshow test, meaning there is no significant discrepancy between the predicted and observed values.

**Table S8. Association between L-Glutamine and Clinical and Radiomics Variables**

| Group | Variable | Beta | SE | t-value | P-value^a^ |
| --- | --- | --- | --- | --- | --- |
| Overall | Maximum2DDiameterSlice | 1.65E-08 | 1.68E-08 | 0.987 | 0.332 |
|  | GrayLevelNonUniformityNormalized | 1.54E-09 | 9.53E-10 | 1.618 | 0.117 |
|  | SizeZoneNonUniformityNormalized | -4.49E-10 | 9.12E-10 | -0.493 | 0.626 |
|  | Glcm_Imc1 | -3.69E-12 | 5.47E-11 | -0.068 | 0.947 |
|  | TG | -7.67E-09 | 7.41E-09 | -1.034 | 0.310 |
|  | TC | -1.30E-08 | 1.20E-08 | -1.083 | 0.288 |
|  | ALT | 5.07E-09 | 1.64E-07 | 0.031 | 0.975 |
|  | AST | 4.92E-08 | 1.46E-07 | 0.338 | 0.738 |
|  | GGT | 4.55E-08 | 1.78E-07 | 0.255 | 0.801 |
|  | ALP | -5.39E-07 | 4.49E-07 | -1.201 | 0.240 |
|  | TBIL | -4.36E-08 | 6.04E-08 | -0.722 | 0.476 |
|  | DBIL | -1.86E-08 | 2.52E-08 | -0.739 | 0.466 |
|  | IBIL | -2.50E-08 | 4.91E-08 | -0.510 | 0.614 |
|  | SCr | -9.41E-08 | 3.76E-07 | -0.250 | 0.804 |
|  | Urea | -2.55E-08 | 1.03E-07 | -0.248 | 0.806 |
|  | L3_subcutaneous_fat_area | 1.23E-05 | 5.98E-05 | 0.205 | 0.839 |
|  | L3_visceral_fat_area | 4.76E-05 | 6.13E-05 | 0.776 | 0.444 |
|  | L3_intermuscular_fat_area | -3.28E-06 | 5.78E-06 | -0.567 | 0.575 |
|  | L3_muscle_area | 1.56E-08 | 2.79E-07 | 0.056 | 0.956 |
| High Risk | Maximum2DDiameterSlice | 2.98E-08 | 2.63E-08 | 1.135 | 0.286 |
|  | GrayLevelNonUniformityNormalized | -3.14E-10 | 2.19E-09 | -0.143 | 0.889 |
|  | SizeZoneNonUniformityNormalized | 3.22E-09 | 2.42E-09 | 1.330 | 0.216 |
|  | Glcm_Imc1 | -1.56E-11 | 7.99E-11 | -0.195 | 0.850 |
|  | TG | 5.11E-09 | 1.04E-08 | 0.493 | 0.634 |
|  | TC | 6.17E-09 | 2.60E-08 | 0.238 | 0.818 |
|  | ALT | 3.54E-07 | 2.64E-07 | 1.337 | 0.214 |
|  | AST | 1.97E-07 | 3.31E-07 | 0.597 | 0.565 |
|  | GGT | 6.63E-07 | 4.38E-07 | 1.514 | 0.164 |
|  | ALP | -1.42E-06 | 6.12E-07 | -2.318 | 0.046 |
|  | TBIL | 1.17E-08 | 1.42E-07 | 0.083 | 0.936 |
|  | DBIL | -4.42E-08 | 5.78E-08 | -0.764 | 0.464 |
|  | IBIL | 5.55E-08 | 1.10E-07 | 0.505 | 0.626 |
|  | SCr | -4.52E-08 | 6.82E-07 | -0.066 | 0.949 |
|  | Urea | 2.27E-07 | 2.65E-07 | 0.857 | 0.413 |
|  | L3_subcutaneous_fat_area | -6.93E-05 | 0.000131 | -0.530 | 0.609 |
|  | L3_visceral_fat_area | -0.00014 | 0.000153 | -0.917 | 0.383 |
|  | L3_intermuscular_fat_area | -3.52E-06 | 8.69E-06 | -0.405 | 0.695 |
|  | L3_muscle_area | -3.24E-07 | 8.25E-07 | -0.393 | 0.704 |
| Low Risk | Maximum2DDiameterSlice | 5.33E-08 | 1.71E-08 | 3.112 | 0.012 |
|  | GrayLevelNonUniformityNormalized | 2.43E-09 | 1.51E-09 | 1.612 | 0.142 |
|  | SizeZoneNonUniformityNormalized | -8.01E-10 | 1.14E-09 | -0.704 | 0.499 |
|  | Glcm_Imc1 | 7.70E-12 | 5.46E-11 | 0.141 | 0.891 |
|  | TG | -9.74E-09 | 1.34E-08 | -0.727 | 0.485 |
|  | TC | -2.51E-08 | 1.87E-08 | -1.341 | 0.213 |
|  | ALT | 5.75E-08 | 1.54E-07 | 0.374 | 0.717 |
|  | AST | -1.20E-07 | 1.79E-07 | -0.671 | 0.519 |
|  | GGT | 5.26E-08 | 1.69E-07 | 0.310 | 0.764 |
|  | ALP | -2.86E-08 | 7.74E-07 | -0.037 | 0.971 |
|  | TBIL | -2.99E-08 | 9.05E-08 | -0.330 | 0.749 |
|  | DBIL | -1.56E-08 | 4.09E-08 | -0.381 | 0.712 |
|  | IBIL | -1.47E-08 | 7.51E-08 | -0.196 | 0.849 |
|  | SCr | 1.04E-07 | 5.47E-07 | 0.190 | 0.853 |
|  | Urea | -2.68E-08 | 9.18E-08 | -0.292 | 0.777 |
|  | L3_subcutaneous_fat_area | 3.86E-05 | 9.17E-05 | 0.421 | 0.684 |
|  | L3_visceral_fat_area | 0.000112 | 8.28E-05 | 1.351 | 0.210 |
|  | L3_intermuscular_fat_area | 2.67E-06 | 6.30E-06 | 0.424 | 0.682 |
|  | L3_muscle_area | 5.69E-08 | 3.12E-07 | 0.183 | 0.859 |

^a^ Comparisons were conducted between the value of L-glutamine and that of each clinical or radiomics variable using multi-variable linear regression. Regression coefficient (Beta) was adjusted for sex, age, BMI, medical history (hypertension, diabetes and thrombosis) and GLIM nutritional status. Beta was considered statistically significant at P < 0.05.

**Appendix S2 Formula of the Radiomics Score and Combined Score**

**Radiomics Score** =

( 0.0432 +

0.3160 * IMG_original_shape_Maximum2DDiameterSlice +

0.9801 * IMG_wavelet-HHH_glszm_GrayLevelNonUniformity -

0.4529 * IMG_wavelet-HHH_glszm_SizeZoneNonUniformityNormalized +

0.2644 * IMG_wavelet-LHH_glcm_Imc1

)

**Combined Score** = -3.8935 + 1.5045 * TC + 1.57 * Urea + 1.5601 * PN_Frequency + 1.3491 * L3_intermuscular_fat_area + 2.4158 * Radiomics_Score

**Appendix S3 Metabolomics Analysis**

**S3.1 Sample Preprocessing**

**S3.1.1 Chemicals and Reagents**

As shown in **Table S9**, LC-MS grade methanol (MeOH) was purchased from Fisher Scientific (Loughborough, UK). 2-Amino-3-(2-chloro-phenyl)-propionic acid was obtained from Aladdin (Shanghai, China). Ultrapure water was generated using a Milli-Q system (Millipore, Bedford, USA).

**S3.1.2 Equipment**

As shown in **Table S10**, High-speed centrifuge was obtained from Hunan Xiangyi Experiment Equipment Co., Ltd. (Hunan, China). Centrifugal vacuum evaporator was from Eppendorf China Ltd. (Shanghai, China). Vortex mixer was obtained from Haimen Kylin-bell Lab Instruments Co., Ltd. (Haimen, China). Microporous membrane filters (0.22 µm) were purchased from Tianjin Jinteng Experiment Equipment Co., Ltd. (Tianjin, China).

**Table S9. The Chemicals and Reagents of Sample Preprocessing**

| Chemicals and Reagents | CAS | Purity | Brand |
| --- | --- | --- | --- |
| methanol | 67-56-1 | ≥99.9% | Thermo |
| 2-Amino-3-(2-chloro-phenyl)-propionic acid | 103616-89-3 | 98% | Aladdin |
| H_2_O | / | / | Millipore |

**Table S10. Equipment of Sample Preprocessing**

| Equipment | Brand | Type |
| --- | --- | --- |
| High-speed centrifuge | Xiangyi | H1850-R |
| Vortex mixer | Kylin-bell | BE-2600 |
| Centrifugal vacuum evaporator | Eppendorf | 5305 |
| Microporous membrane filters | Jinteng | - 1. m PTFE |

**S3.1.3 Sample Preparation [1]**

(1) Thaw the experimental sample at 4℃, vortex the sample for 1 min after thawing, and mix evenly;

(2) Accurately transfer an appropriate amount of sample into a 2 mL centrifuge tube;

Add 400 µL methanol and vortex for 1 min;

(3) Centrifuge for 10 min at 12,000 rpm and 4 °C, take all the supernatant transfer it to a new 2 mL centrifuge tube, concentrate and dry it;

(4) Add 150 µL of 2-chloro-l-phenylalanine (4 ppm) solution prepared with 80% methanol water to redissolve the sample, filter the supernatant by 0.22 μm membrane and transfer into the detection bottle for LC-MS detection.

**S3.2 Data Acquisition**

**S3.2.1 Reagents and Equipment**

As shown in **Table S11**, LC-MS grade acetonitrile (ACN) was purchased from Fisher Scientific (Loughborough, UK). Formic acid was obtained from TCI (Shanghai, China). Ammonium formate was obtained from Sigma-Aldrich (Shanghai, China). Ultrapure water was generated using a Milli-Q system (Millipore, Bedford, USA).

**S3.2.2 Liquid Chromatography Conditions**

As shown in **Table S12**, The LC analysis was performed on a Vanquish UHPLC System (Thermo Fisher Scientific, USA). Chromatography was carried out with an ACQUITY UPLC ® HSS T3 (2.1×100 mm, 1.8 µm) (Waters, Milford, MA, USA). The column maintained at 40 ℃. The flow rate and injection volume were set at 0.3 mL/min and 2 μL, respectively. For LC-ESI (+)-MS analysis, the mobile phases consisted of (B2) 0.1% formic acid in acetonitrile (v/v) and (A2) 0.1% formic acid in water (v/v). Separation was conducted under the following gradient: 0~1 min，10% B2；1~5 min，10%~98% B2；5~6.5 min，98% B2；6.5~6.6 min，98%~10% B2；6.6~8 min，10% B2. For LC-ESI (-)-MS analysis, the analytes was carried out with (B3) acetonitrile and (A3) ammonium formate (5mM). Separation was conducted under the following gradient: 0~1 min，10% B3；1~5 min，10%~98% B3；5~6.5 min，98% B3；6.5~6.6 min，98%~10% B3；6.6~8 min，10% B3 [2].

**S3.2.3 Mass Spectrum Conditions**

As shown in **Table S12**, Mass spectrometric detection of metabolites was performed on Orbitrap Exploris 120 (Thermo Fisher Scientific, USA) with ESI ion source. Simultaneous MS1 and MS/MS (Full MS-ddMS2 mode, data-dependent MS/MS) acquisition was used. The parameters were as follows: sheath gas pressure, 40 arb; aux gas flow, 10 arb; spray voltage, 3.50 kV and -2.50 kV for ESI(+) and ESI(-), respectively; capillary temperature, 325 ℃; MS1 range, m/z 100-1000; MS1 resolving power, 60000 FWHM; number of data dependant scans per cycle, 4; MS/MS resolving power, 15000 FWHM; normalized collision energy, 30%; dynamic exclusion time, automatic [3].

**Table S11. The Chemicals and Reagents of Metabolomic Analysis**

| Chemicals and Reagents | CAS | Purity | Brand |
| --- | --- | --- | --- |
| acetonitrile | 75-05-8 | ≥99.9% | Thermo |
| Formic acid | 64-18-6 | LC-MS grade | TCI |
| Ammonium formate | 540-69-2 | ≥99.9% | Sigma |
| H_2_O | / | / | Millipore |

**Table S12. Equipment of Metabolomic Analysis**

| Equipment | Brand | Type |
| --- | --- | --- |
| Liquid chromatograph | Thermo | Vanquish |
| Mass spectrometer | Thermo | Orbitrap Exploris 120 |

**S3.3 Data Preprocessing**

The raw data were firstly converted to mzXML format by MSConvert in ProteoWizard software package (v3.0.8789) [4] and processed using R XCMS(v3.12.0) for feature detection [5], retention time correction and alignment. Key parameters settings were set as follows: ppm=15, peakwidth=c(5, 30), mzdiff=0.01, method=centWave. The batch effect was then eliminated by correcting the data based on QC samples. Metabolites with RSD > 30% in QC samples were filtered and then used for subsequent data analysis. The metabolites were identified by accuracy mass and MS/MS data which were matched with HMDB (<http://www.hmdb.ca>) [6], massbank (<http://www.massbank.jp/>) [7], KEGG (<https://www.genome.jp/kegg/>) [8], LipidMaps (<http://www.lipidmaps.org>) [9], mzcloud (<https://www.mzcloud.org>) [10] and the metabolite database bulid by Panomix Biomedical Tech Co., Ltd. (Shuzhou, China). The molecular weight of metabolites was determined according to the m/z (mass-to-charge ratio) of parent ions in MS data. Molecular formula was predicted by ppm (parts per million) and adduct ion, and then matched with the database. At the same time, the MS/MS data from quantitative table of MS/MS data, were matched with the fragment ions and other information of each metabolite in the database, so as to realize the MS/MS identification of metabolites.

**S3.4 Data Analysis**

Two different multivariate statistical analysis models, unsupervised and supervised, were applied to discriminate the groups (PCA; PLS-DA; OPLS-DA) by R ropls (v1.22.0) package [11]. The statistical significance of P.value was obtained by statistical test between groups. Finally, combined with P.value, VIP (OPLS-DA variable projection importance) and FC (multiple of difference between groups) to screen biomarker metabolites. By default, when P value < 0.05 and VIP value > 1, we think that metabolite was considered to have significant differential expression.

**S3.5 Pathway Analysis**

Differential metabolites were subjected to pathway analysis by MetaboAnalyst [12]^,^ which combines results from powerful pathway enrichment analysis with the pathway topology analysis. The identified metabolites in metabolomics were then mapped to the KEGG pathway for biological interpretation of higher-level systemic functions. The metabolites and corresponding pathways were visualized using KEGG Mapper tool.

**Reference**

[1] Demurtas A, Pescina S, Nicoli S, Santi P, Ribeiro De Araujo D, Padula C. Validation of a HPLC-UV method for the quantification of budesonide in skin layers. J Chromatogr B Analyt Technol Biomed Life Sci 2021;1164:122512-122512.

[2] Zelena E, Dunn WB, Broadhurst D, Francis-Mcintyre S, Carroll KM, Begley P, et al. Development of a Robust and Repeatable UPLC−MS Method for the Long-Term Metabolomic Study of Human Serum. Anal Chem 2009;81:1357-1364.

[3] Want EJ, Masson P, Michopoulos F, Wilson ID, Theodoridis G, Plumb RS, et al. Global metabolic profiling of animal and human tissues via UPLC-MS. Nat Protoc 2013;8:17-32.

[4] Rasmussen JA, Villumsen KR, Ernst M, Hansen M, Forberg T, Gopalakrishnan S, et al. A multi-omics approach unravels metagenomic and metabolic alterations of a probiotic and synbiotic additive in rainbow trout (Oncorhynchus mykiss). Microbiome 2022;10:21-19.

[5] Navarro-Reig M, Jaumot J, García-Reiriz A, Tauler R. Evaluation of changes induced in rice metabolome by Cd and Cu exposure using LC-MS with XCMS and MCR-ALS data analysis strategies. Anal Bioanal Chem 2015;407:8835-8847.

[6] Wishart DS, Tzur D, Knox C, Eisner R, Guo AC, Young N, et al. HMDB: the Human Metabolome Database. Nucleic Acids Res 2007;35:D521-D526.

[7] Horai H, Arita M, Kanaya S, Nihei Y, Ikeda T, Suwa K, et al. MassBank: a public repository for sharing mass spectral data for life sciences. Journal of mass spectrometry. 2010;45:703-714.

[8] Ogata H, Goto S, Sato K, Fujibuchi W, Bono H, Kanehisa M. KEGG: Kyoto Encyclopedia of Genes and Genomes. Nucleic Acids Res 1999;27:29-34.

[9] Sud M, Fahy E, Cotter D, Brown A, Dennis EA, Glass CK, et al. LMSD: LIPID MAPS structure database. Nucleic Acids Res 2007;35:D527-32.

[10] Abdelrazig S, Safo L, Rance GA, Fay MW, Theodosiou E, Topham PD, et al. Metabolic characterisation of Magnetospirillum gryphiswaldense MSR-1 using LC-MS-based metabolite profiling. RSC Adv 2020;10:32548-32560.

[11] Thévenot EA, Roux A, Xu Y, Ezan E, Junot C. Analysis of the Human Adult Urinary Metabolome Variations with Age, Body Mass Index, and Gender by Implementing a Comprehensive Workflow for Univariate and OPLS Statistical Analyses. J Proteome Res 2015;14:3322-3335.

[12] Xia J, Wishart DS. Web-based inference of biological patterns, functions and pathways from metabolomic data using MetaboAnalyst. Nat Protoc 2011;6:743-760.
